# Design, Synthesis, and Biological Evaluation of a Novel Aminothiol Compound as Potential Radioprotector

**DOI:** 10.1155/2021/4714649

**Published:** 2021-08-21

**Authors:** Xuejiao Li, Xinxin Wang, Longfei Miao, Yuying Guo, Renbin Yuan, Jingming Ren, Yichi Huang, Hongqi Tian

**Affiliations:** Tianjin Key Laboratory of Radiation Medicine and Molecular Nuclear Medicine, Institute of Radiation Medicine, Peking Union Medical College and Chinese Academy of Medical Sciences, Tianjin 300192, China

## Abstract

The risk of radiation damage has increased with the rapid development of nuclear technology and radiotherapy. Hence, research on radioprotective agents is of utmost importance. In the present study, a novel aminothiol compound 12, containing a linear alkylamino backbone and three terminal thiols, was synthesized. Owing to the appropriate capped groups in the chains, it has an improved permeability and oral bioavailability compared to other radioprotective agents. Oral administration of compound 12 improved the survival of mice that received lethal doses of *γ*-irradiation. Experimental results demonstrated that compound 12 not only mitigated total body irradiation-induced hematopoietic injury by increasing the frequencies of hematopoietic stem and progenitor cells but also prevented abdominal irradiation-induced intestinal injury by increasing the survival of Lgr5^+^ intestinal cells, lysozyme^+^ Paneth cells, and Ki67^+^ cells. In addition, compound 12 decreased oxidative stress by upregulating the expression of Nrf2 and NQO1 and downregulating the expression of NOX1. Further, compound 12 inhibited *γ*-irradiation-induced DNA damage and alleviated G2/M phase arrest. Moreover, compound 12 decreased the levels of p53 and Bax and increased the level of Bcl-2, demonstrating that it may suppress radiation-induced apoptosis via the p53 pathway. These results indicate that compound 12 has the possibility of preventing radiation injury and can be a potential radioprotector for clinical applications.

## 1. Introduction

Humans may be exposed to ionizing radiation (IR) during radiological attacks and accidents, exploration of the universe, and radiation therapy [1–3]. Under these cases, IR can cause damage to macromolecules in cells through two mechanisms: direct action, in which direct deposition of radiant energy on biological molecules cause their ionization or excitation, or the indirect action, in which radiant energy acts on water molecules and produces reactive oxygen species (ROS), which can disrupt the structure of biomolecules [4]. Hence, IR can attack DNA and proteins, ultimately inducing cell death and organ dysfunction [5, 6]. It has been reported that the hematopoietic system and gastrointestinal (GI) tissues are especially sensitive to IR; hence, it is extremely essential to develop countermeasures to protect these tissues from radiation injury [7, 8].

Radioprotectors are compounds used before or during radiation exposure to prevent radiation damage [9]. Over the past few decades, a multitude of radioprotectors have been developed, including amifostine, 2,2-dimethylthiazolidine, PrC-210, glutathione, and resveratrol [10, 11]. And aminothiol radioprotectors have gained attention because of their ability to rapidly improve the resistance of cells to radiation exposure as well as their capacity to protect against high levels of radiation [12]. Aminothiol compounds contain an alkylamino backbone and thiol groups [13]. Because of the amine groups in the alkyl backbone, they carry a positive charge so that they can interact with negatively charged DNA in cells, and the thiol groups can scavenge free radicals generated by IR [13]. To date, the most effective aminothiol radioprotector is amifostine [8]. However, it has a narrow preexposure time window and can only be administered via intravenous injection [14]. Moreover, it can induce side effects such as vomiting, hypotension, hypocalcemia, and allergic reactions [15, 16]. Owing to these drawbacks, the clinical application of amifostine has been significantly limited.

In the current study, we designed and synthesized a novel aminothiol compound. It contains a linear alkylamino backbone and three thiols which are capped at the ends of the alkyl side chains. Compared with the aminothiol compounds reported in previous studies [10, 13, 17], it has the following merits: (1) it contains more thiols that can exert ROS scavenging effects, (2) the thiol groups in the molecule are placed away from the alkyl backbone to enable the scavenging of ROS before they attack the DNA molecules; and (3) the amine group and the thiols in the compound are capped to increase its stability and permeability, for better absorption when administered orally. After obtaining the target compound, we investigated its radioprotective activity and mechanisms of action. Our results showed that it could prevent total body irradiation- (TBI-) induced bone marrow injury and alleviate abdominal irradiation- (ABI-) induced intestinal injury by reducing oxidative stress and inhibiting apoptosis. These results provide new ideas for the further design and development of radioprotectors.

## 2. Materials and Methods

### 2.1. Synthesis of the Target Compound 12

**Scheme 1:**
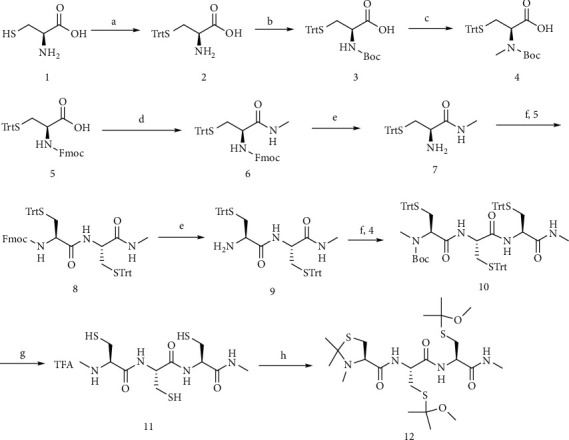
Synthesis of compound 12. Reagents and conditions: (a) TrtCl/DMF, 65°C; (b) Boc_2_O, NaOH/dioxane, 0–5°C; (c) MeI, NaH/THF; (d) MeNH_2_, CDI/THF, 0–5°C; (e) piperidine/DMF; (f) HOBt, EDCI/DCM; (g) TFA, TIPS/DCM; (h) 2,2-dimethoxypropane, acetone.

Compound 12 was synthesized as described in Scheme 1. N-(tert-Butoxycarbonyl)-S-trityl-L-cysteine (3) was obtained in high yield following the conventional approach of protecting group. Compound 3 was methylated with iodomethane to get compound 4 in moderate yield. (R)-2-Amino-N-methyl-3-(tritylthio) propenamide (7) was synthesized from N-(((9H-fluoren-9-yl) methoxy) carbonyl)-S-trityl-L-cysteine (5) using condensation and deprotection reactions. Compound 8 was prepared through the condensation of compound 7 and compound 5 in the presence of HOBt and EDCI. Compound 8 was deprotected with piperidine in DMF to produce compound 9, which was then reacted with compound 4 to obtain compound 10. Compound 11 was prepared via quantitative deprotection from compound 10. It was then protected by 2,2-dimethoxypropane to give compound 12. A detailed procedure for the preparation of compound 12 is provided in Supplementary Materials.

### 2.2. Animals, Irradiation, and Treatments

Male C57BL/6 mice (6–8 weeks old) were obtained from HFK Bioscience Co. Inc. (Beijing) and bred in a pathogen-free environment at the laboratory animal center of the Institute of Radiation Medicine (IRM), Chinese Academy of Medical Sciences (CAMS). All animal experiments were performed according to the NIH *Guide for the Care and Use of Laboratory Animals* and were permitted by the Institutional Animal Care and Use Committee of the IRM, CAMS.

Irradiation was carried out using a ^137^Cs *γ*-ray source placed in the Exposure Instrument Gammacell-40 (Atomic Energy of Canada Ltd., Ontario, Canada) at the dose rate of 0.99 Gy/min. For the TBI study, mice were randomly divided into five groups in the survival experiment: control, 8 Gy + vehicle, 8 Gy + 12 (900 mg/kg), 8 Gy + 12 (1200 mg/kg), and 8 Gy + 12 (1500 mg/kg) (*n* = 10 each). In the remaining TBI studies, mice were randomly divided into four groups: control, 12 (1200 mg/kg), 4 Gy, and 4 Gy + 12 (1200 mg/kg) (*n* = 5 each). For the ABI experiments, a lead shield was used such that the entire abdomen was irradiated and other parts were shielded, and the mice were assigned to three groups: control, 15 Gy + vehicle, and 15 Gy + 12 (1200 mg/kg) (*n* = 10 or *n* = 5). The mice in the IR + 12 groups received compound 12 via oral gavage 1 h before irradiation. The mice in other groups were treated with vehicle or compound 12 in the same way.

### 2.3. Survival Experiments

Mice were exposed to TBI or ABI at the indicated doses. The status of the mice was monitored for 30 days following radiation, and data are presented as survival rates.

### 2.4. Analysis of Peripheral Blood

For hematopoietic damage experiments, the mice in the TBI groups received 4.0 Gy *γ*-irradiation and were sacrificed on day 14 after irradiation. Peripheral blood was collected from the orbital sinus. White blood cells (WBCs), red blood cells (RBCs), platelets (PLTs), hemoglobin (HGB), the percentage of lymphocytes (LY%), and the percentage of neutrophils (NE%) were analyzed using a hematology analyzer (Nihon Kohden, Japan).

### 2.5. Flow Cytometry Analysis of Hematopoietic Stem and Progenitor Cells (HSPCs)

For analysis of hematopoietic stem cells (HSCs) and hematopoietic progenitor cells (HPCs), bone marrow cells were isolated from mouse femurs with sterile PBS and filtered. Lineage^−^Sca1^+^c-kit^+^ (LSK) was employed to define HSCs as suggested in earlier studies [3, 18, 19]. Bone marrow cells (5 × 10^6^) were first labeled with biotin-conjugated antibodies recognizing Gr1, CD4, CD8, CD11b, B220, and Ter119 (mixed lineage antibodies, BioLegend, CA, USA) and then incubated with streptavidin-PerCP (BioLegend), sca1-PE, and c-kit-APC antibodies (eBioscience, CA, USA). The frequencies of HPCs and HSCs were determined via flow cytometry and analyzed by using BD Accuri C6 software (BD Biosciences, USA).

### 2.6. Evaluation of Intracellular ROS Levels

To evaluate ROS levels, 5 × 10^6^ bone marrow cells were incubated with LSK and HPC antibodies as described above and then stained with 2′,7′-dichlorodihydrofluorescein diacetate (DCFH-DA) at a concentration of 10 *μ*M for 25 min at 37°C. Intracellular ROS levels were evaluated by determining the mean fluorescence intensity (MFI) of DCFH-DA using flow cytometry.

### 2.7. Histological Analysis

For intestinal injury experiments, mice in ABI groups received irradiation (15.0 Gy dose), and all mice were sacrificed on day 5 after irradiation. The small intestine of each mouse was collected, fixed in 4% formaldehyde, dehydrated, and embedded in paraffin. Next, the blocks were cut into 4 *μ*m thick sections. The paraffin sections were deparaffinized, rehydrated, and stained with hematoxylin and eosin (HE). Images were acquired using a microscope (Olympus, Tokyo, Japan). For histological analysis, five circular fields were analyzed per mouse in a blind fashion to count the number of crypts and measure the length of the villi.

### 2.8. Immunohistochemistry Analysis

Sections of the small intestine (4 *μ*m thick) were deparaffinized and rehydrated. To block endogenous peroxidase, deparaffinized sections were incubated in 3% hydrogen peroxide for 15 min at 37°C. Then, antigen retrieval was performed by boiling the sections in a citrate buffer solution, and the sections were blocked with goat serum. Next, these sections were incubated with anti-Villin antibody (1 : 100 dilution, Bioworld, MN, USA), anti-Lgr5 antibody (1 : 200 dilution, Bioworld), anti-lysozyme antibody (1 : 100 dilution, Bioworld), or anti-Ki67 antibody (1 : 200 dilution, Abcam, Cambridge, UK) overnight at 4°C. The sections were then stained with the corresponding secondary antibodies for 1 h at 37°C. Finally, the DAB kit was used to detect the cells, and the sections were stained with hematoxylin. Images were acquired using the microscope (Olympus), and immunoreactive cells were analyzed in a blind fashion using the ImageJ software.

### 2.9. TUNEL Assay

The 4 *μ*m thick small intestine sections were deparaffinized, rehydrated, and incubated with proteinase K for 20 minutes at 37°C. The sections were stained with a TUNEL mix at 37°C for 1 h. Next, Converter-POD was added, and the samples were incubated at 37°C for 30 min. After that, the DAB kit was used to detect the cells, and the sections were counterstained with hematoxylin. Ultimately, images were obtained using the microscope (Olympus).

### 2.10. Immunofluorescence Analysis

The 4 *μ*m thick small intestine sections were deparaffinized and rehydrated, and antigen retrieval was performed as described above. The sections were blocked with goat serum at 37°C for 1 h and incubated with anti-*γ*-H2AX antibody (1 : 400 dilution, Bioworld), anti-p53 antibody (1 : 100 dilution, Bioworld), anti-caspase-9 antibody (1 : 50 dilution, CST, MA, USA), or anti-caspase-3 antibody (1 : 500 dilution, CST) overnight at 4°C. The sections were then incubated with the corresponding secondary antibodies for 40 min at 37°C in darkness. Ultimately, the sections were sealed with DAPI-containing mounting medium, and the fluorescence microscope (Olympus) was used to capture images.

### 2.11. Cell Culture

Rat intestinal epithelial cell 6 (IEC-6) was cultured in DMEM medium, which contains 10% fetal bovine serum, penicillin (100 U/mL), streptomycin (100 *μ*g/mL), and bovine insulin (10 *μ*g/mL). The cells were kept in a humid atmosphere containing 5% CO_2_ at 37°C. The ^137^Cs *γ*-ray source was used for subsequent studies at the dose rate of 0.99 Gy/min.

### 2.12. Western Blotting

IEC-6 cells were seeded in 6-well plates (4 × 10^5^ cells per well) and treated with compound 12 (100 *μ*M) for 2 h before irradiation (8.0 Gy dose). After culturing for 12 or 24 h, the cells were harvested, and the nuclear and cytoplasmic proteins were extracted using a nuclear/cytoplasmic protein extraction kit (Solarbio, Beijing, China). Proteins were isolated on SDS–PAGE gels and transferred to polyvinylidene difluoride (PVDF) membranes. After blocking with 10% skim milk, the membranes were incubated at 4°C overnight with antibodies against p53 (1 : 3000 dilution, Proteintech, Wuhan, China), nuclear factor erythroid 2-related factor 2 (Nrf2, 1 : 1000 dilution, Proteintech), Bax (1 : 5000 dilution, Abcam), Bcl-2 (1 : 1000 dilution, Abcam), p21 (1 : 1000 dilution, Abcam), NADPH quinone oxidoreductase 1 (NQO1, 1 : 25000 dilution, Proteintech), NADPH oxidase 1 (NOX1, 1 : 1000 dilution, Proteintech), Lamin B1 (1 : 5000 dilution, Proteintech), and GAPDH (1 : 20000 dilution, Proteintech). Next, the membranes were stained with the appropriate secondary antibodies at room temperature for 1 h. The bands were developed using enhanced chemiluminescence reagent and analyzed by using Image Lab™ software (Bio-Rad, USA).

### 2.13. Flow Cytometry Analysis of Cell Cycle Distribution

IEC-6 cells were seeded in 6-well plates at 3 × 10^5^ cells per well and treated with compound 12 (100 *μ*M) for 2 h before irradiation (8.0 Gy dose). After incubation for 12 h, the cells were collected and washed with precooled PBS. Next, the cells were fixed with 70% ethanol at 4°C overnight. After washing with PBS, the cells were stained with propidium iodide (PI) staining buffer (10 *μ*L PI, 10 *μ*L RNase A, and 0.5 mL staining solution per sample) for 30 min at 37°C. Finally, the samples were analyzed using flow cytometry, and the cell cycle distribution was analyzed using ModFit LT 4.1 software.

### 2.14. Immunofluorescence Staining

IEC-6 cells were plated in 12-well plates with cell slides (5 × 10^4^ cells per well). The cells were pretreated with compound 12 (100 *μ*M) for 2 h before irradiation (8.0 Gy dose). After incubation for 24 h, the cells were fixed with 4% paraformaldehyde for 20 min and permeabilized in 0.3% Triton X-100 for 30 min. The slides were then blocked with 2% BSA, stained with antibody recognizing Nrf2 (1 : 100 dilution, Proteintech) for 1.5 h at room temperature, and incubated with PE-labeled secondary antibody (1 : 100 dilution, Proteintech) for 1.5 h. Finally, the slides were sealed with DAPI-containing mounting medium, and images were obtained using the fluorescence microscope (Olympus).

### 2.15. Statistical Analysis

For the analysis of survival rates, the Kaplan-Meier method and log-rank test were employed in the present study. An unpaired *t*-test (two-tailed) was employed for comparisons of the mean, and data are expressed as the mean ± SEM. Statistical analysis was carried out using GraphPad Prism 5 software. And statistical significance was set at *p* < 0.05.

## 3. Results

### 3.1. Synthesis and Characterizations of Compound 12

The synthesis of compound 12 is illustrated in Scheme 1. All intermediates and the final product were synthesized as presented in Supplementary Materials. The compounds were characterized by ^1^H NMR (Figures S4–S13). And the target compound 12 was also characterized by ^13^C NMR and HRMS (Figures S14 and S15). Compound 12 (purity > 95%) was used in all the experiments (Figure S16).

### 3.2. Compound 12 Improved the Survival Rate of Mice after TBI or ABI

**Figure 1:**
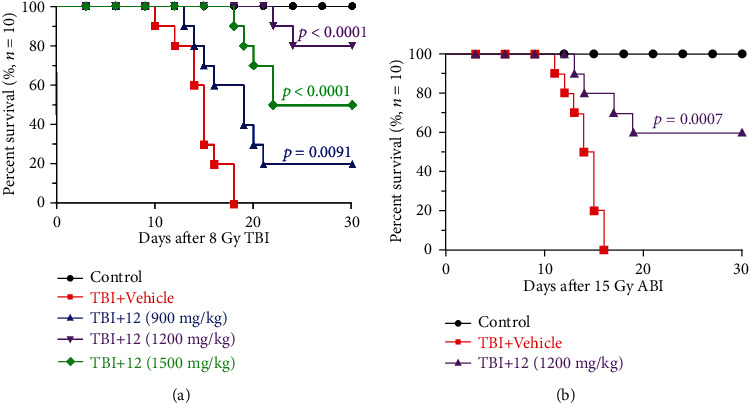
Compound 12 improved the survival of mice after lethal doses of IR. Survival analysis of mice receiving (a) TBI (8.0 Gy dose) and (b) ABI (15.0 Gy dose) is shown (*n* = 10).

To determine the effect of compound 12 on TBI-induced fatal damage in mice, we investigated the survival rate of mice receiving a lethal dose (8.0 Gy) of irradiation [20]. As demonstrated in Figure 1(a), three doses of compound 12 improved the survival of mice in comparison to the TBI+vehicle group (*p* < 0.05): the survival rate of the mice in the 1200 mg/kg group was 80%, while that in the 1500 mg/kg group was 50%. We speculated that a low dose of compound 12 would not be effective, and a high dose of compound 12 might exert toxic effects. Therefore, compound 12 was administered at a dose of 1200 mg/kg in the following animal studies.

To assess the effect of compound 12 on ABI-induced fatal intestinal damage in mice, we studied their survival rate after a lethal dose (15.0 Gy) of irradiation [21]. As demonstrated in Figure 1(b), the survival rate of the mice pretreated with compound 12 (1200 mg/kg) increased by 60% (*p* = 0.0007) compared to the ABI+vehicle group. These results demonstrate that compound 12 can improve the survival of mice receiving a lethal dose of ABI.

### 3.3. Compound 12 Alleviated TBI-Induced Hematopoietic Damage

**Figure 2:**
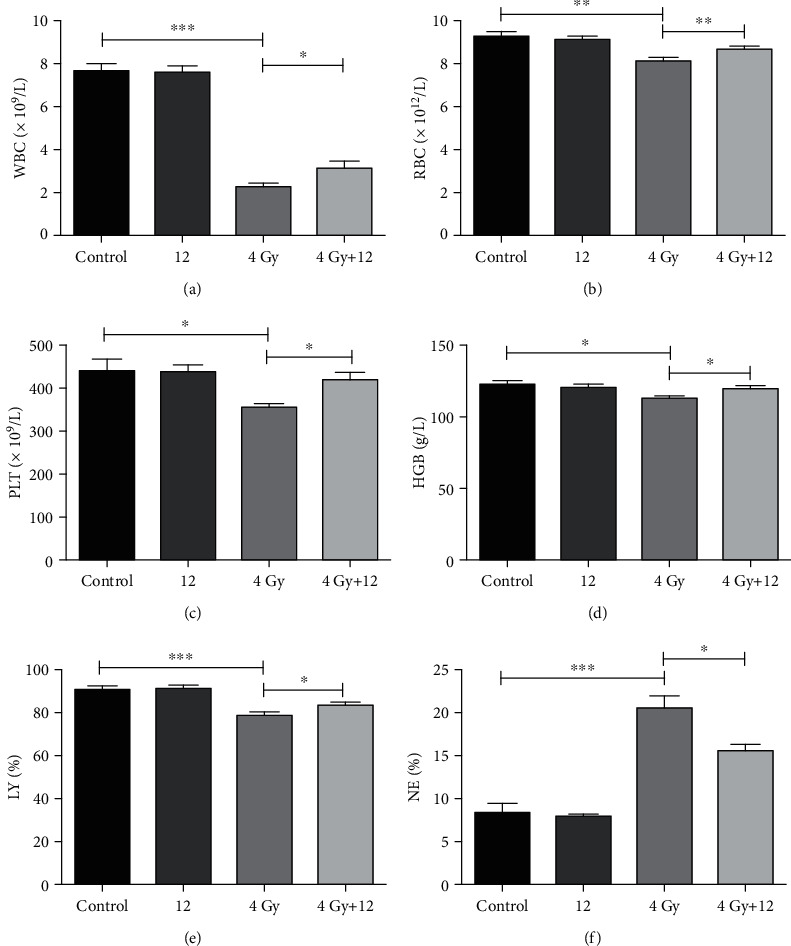
Compound 12 alleviated hematopoietic injury in TBI-exposed mice. (a) WBCs, (b) RBCs, (c) PLTs, (d) HGB, (e) LY%, and (f) NE% in peripheral blood were analyzed on the 14th day after TBI. ^∗^*p* < 0.05, ^∗∗^*p* < 0.01, and ^∗∗∗^*p* < 0.001; *n* = 5.

Myelosuppression, which manifests as a remarkable reduction of peripheral blood cells, has been considered a classical symptom of TBI-induced hematopoietic damage [22]. Lymphoid-biased HSCs were reported to be more sensitive to irradiation than myeloid-biased HSCs, which could result in myeloid skewing in mice that receive TBI [21, 23]. As shown in Figures 2(a)–2(f), WBCs, RBCs, PLTs, HGB, and LY% in the peripheral blood of the mice that received a sublethal dose (4.0 Gy) of TBI [20] decreased significantly compared to those in the control group, whereas NE% in the peripheral blood of irradiated mice increased dramatically (*p* < 0.05). Nevertheless, treatment with compound 12 markedly increased the abundance of WBCs, RBCs, PLTs, HGB, and LY and decreased that of NE in the peripheral blood, suggesting that it could effectively protect against hematopoietic injury in TBI-exposed mice.

### 3.4. Compound 12 Mitigated Bone Marrow Hematopoietic Cell Damage in TBI-Exposed Mice

**Figure 3:**
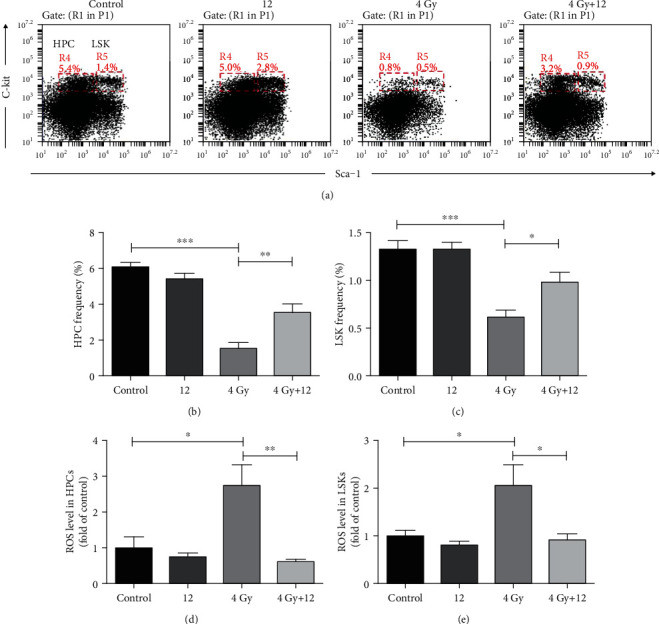
Compound 12 alleviated TBI-induced hematopoietic tissue damage. (a) Representative FACS plots showing the frequencies of HPCs and LSKs. The proportions of (b) HPCs and (c) LSKs in lineage^−^ cells and the levels of ROS in (d) HPCs and (e) LSKs were analyzed. ^∗^*p* < 0.05, ^∗∗^*p* < 0.01, and ^∗∗∗^*p* < 0.001; *n* = 5.

Exposure to IR may induce hematopoietic system damage and HSPC depletions. To determine the role of compound 12 in the changes of bone marrow hematopoietic cells, we explored the effects of compound 12 on the proportions of LSKs and HPCs in the mouse bone marrow (Figure 3(a)). As illustrated in Figures 3(b) and 3(c), the frequencies of LSKs and HPCs in the mice of the TBI+vehicle group were significantly lower than those in the control mice (*p* < 0.001). Nevertheless, compound 12 notably increased the frequencies of LSKs and HPCs. These findings demonstrated that compound 12 could protect mice from TBI-induced HSPC damage.

### 3.5. Compound 12 Reduced Oxidative Stress in the Hematopoietic Cells of TBI-Exposed Mice

Oxidative stress induced by ROS accumulation after radiation exposure can contribute to severe tissue damage [3]. As shown in Figures 3(d) and 3(e), compared with the control group, the ROS levels in HPCs and LSKs of TBI-exposed mice increased markedly (*p* < 0.05), and compound 12 significantly reduced the ROS levels in HPCs and LSKs (*p* < 0.05). These findings indicate that compound 12 might mitigate radiation-induced oxidative stress damage by reducing ROS levels in HSPCs.

### 3.6. Compound 12 Alleviated Structural Damages of Mouse Small Intestine after ABI

**Figure 4:**
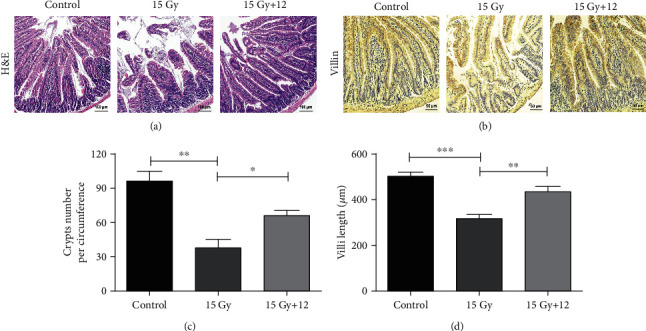
Compound 12 reduced the structural damages of the small intestines following ABI exposure. (a) Representative H&E-stained small intestinal sections. Scale bar: 100 *μ*m. (b) Immunohistochemistry images exhibiting the expression of Villin. Scale bar: 50 *μ*m. Quantitative analysis of (c) crypt number and (d) villi length based on H&E-stained sections is presented. ^∗^*p* < 0.05, ^∗∗^*p* < 0.01, and ^∗∗∗^*p* < 0.001; *n* = 5.

Ionizing radiation can induce the apoptosis of intestinal epithelial cells and cause the breakdown of the intestinal mucosal barrier [24, 25]. As shown in Figures 4(a), 4(c), and 4(d), on day 5 after receiving 15.0 Gy ABI, the crypt number and villi length of the mice were significantly reduced compared with that of the control (*p* < 0.05). In comparison to the mice in the ABI+vehicle group, mice in the ABI + 12 group exhibited more crypts and increased villi length (*p* < 0.05). In addition, IR notably reduced the expression of Villin in intestinal cells, whereas compound 12 significantly increased its expression (Figure 4(b)). These data indicate that the villus-crypt structures of the small intestine in ABI-exposed mice can be well-preserved after treatment with compound 12.

### 3.7. Compound 12 Improved the Regenerative and Differentiative Capacity of Intestinal Stem Cells (ISCs) after ABI

**Figure 5:**
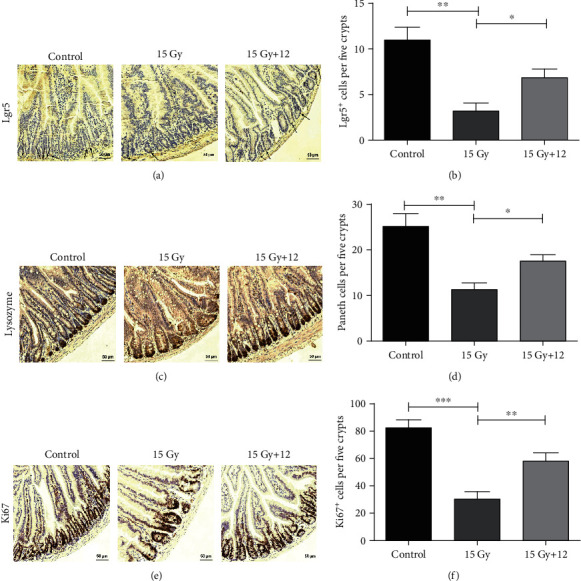
Compound 12 improved the regenerative and differentiative capacity of ISCs following ABI exposure. Immunohistochemistry images exhibit the expressions of (a) Lgr5, (c) lysozyme, and (e) Ki67 in the small intestines. Scale bar: 50 *μ*m. Quantifications of (b) Lgr5^+^ cells, (d) Paneth cells, and (f) Ki67^+^ cells are displayed. ^∗^*p* < 0.05, ^∗∗^*p* < 0.01, and ^∗∗∗^*p* < 0.001; *n* = 5.

The intestinal epithelium undergoes continual self-renewal to maintain homeostasis and has a considerable capacity for regeneration after injuries [26–28]. Lgr5 is a marker of mitotically active cells in the small intestine, and Lgr5^+^ ISCs are crucial for intestinal regeneration after exposure to IR [29–31]. Paneth cells secrete lysozyme, which is essential for maintaining intestinal homeostasis [32]. Furthermore, Ki67 is a well-established marker of epithelial regeneration [33–35]. We investigated the effects of compound 12 on the proliferation and differentiation of intestinal epithelial cells by analyzing Lgr5^+^ ISCs, lysozyme^+^ Paneth cells, and Ki67^+^ cells by immunohistochemistry methods. As shown in Figures 5(a)–5(f), compared with the control, the survivals of Lgr5^+^ cells, Paneth cells, and Ki67^+^ cells were significantly decreased after ABI (*p* < 0.01), but the numbers of these cells in the ABI+12 group were obviously increased (*p* < 0.05). These results clearly demonstrate that compound 12 is helpful in improving the regenerative and differentiative capacity of ISCs in irradiated mice.

### 3.8. Compound 12 Alleviated DNA Injury in the Mouse Small Intestines after ABI

**Figure 6:**
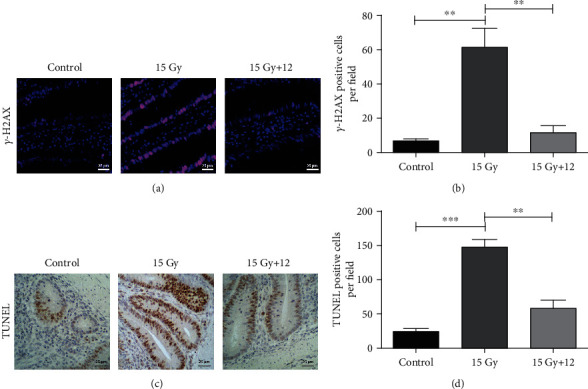
Compound 12 reduced DNA damage and apoptosis in ABI-exposed mice. (a) Representative images showing the expression of *γ*-H2AX in the sections (red: *γ*-H2AX; blue: DAPI). (b) Quantification of *γ*-H2AX-positive cells in the sections. (c) Representative TUNEL-stained small intestinal sections. (d) Statistical analysis of TUNEL-positive cells in the sections. Data are presented as the mean ± SEM (*n* = 5). ^∗∗^*p* < 0.01, ^∗∗∗^*p* < 0.001. Scale bar: 25 *μ*m.

Ionizing radiation can cause DNA damage, especially DNA double-strand breaks (DSBs). Phosphorylation of histone H2AX at serine 139 (*γ*-H2AX) has been regarded as a marker of DSBs [36]. Therefore, we used immunofluorescence staining to investigate the expression of *γ*-H2AX in the small intestine of mice. As shown in Figures 6(a) and 6(b), compared to the control, the expression of *γ*-H2AX in the mouse small intestine increased significantly after ABI, and treatment with compound 12 obviously reduced its expression (*p* < 0.01). This result indicated that compound 12 could alleviate irradiation-induced DNA damage in mice.

### 3.9. Compound 12 Inhibited IR-Induced Apoptosis via the Regulation of p53-Dependent Apoptotic Pathway

IR can induce tissue damage by promoting apoptosis. We assessed apoptosis in mouse small intestines using the TUNEL assay. As illustrated in Figures 6(c) and 6(d), compared to the control, there were more apoptotic cells in irradiated mice (*p* < 0.001), while compound 12 markedly reduced the number of apoptotic cells (*p* < 0.01).

**Figure 7:**
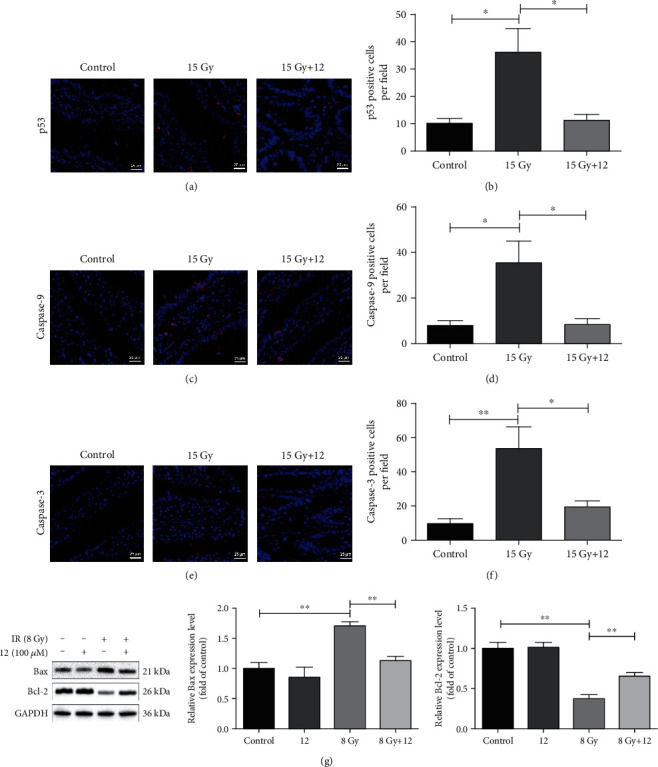
Compound 12 inhibited IR-induced apoptosis through the p53 pathway. Representative images show the expression of (a) p53, (c) caspase-9, and (e) caspase-3 in small intestinal sections (blue: DAPI; red: corresponding proteins). Scale bar: 25 *μ*m. Quantifications of (b) p53 positive, (d) caspase-9 positive, and (f) caspase-3 positive cells are displayed (*n* = 5). (g) Analysis of Bax and Bcl-2 expression in the cytoplasm of IEC-6 cells via Western blotting. Cells were cultured for 24 h after irradiation; data are expressed as the mean ± SEM (*n* = 3). ^∗^*p* < 0.05, ^∗∗^*p* < 0.01.

Furthermore, we explored the role of compound 12 in IR-induced cell apoptosis using immunofluorescence staining and Western blotting. As displayed in Figures 7(a)–7(f), compared to the control, the expression of p53, caspase-9, and caspase-3 in the small intestinal sections was dramatically increased after ABI exposure. However, in the intestines of mice pretreated with compound 12, their expression levels decreased obviously (*p* < 0.05). Furthermore, as described in Figure 7(g), the level of Bax in IEC-6 cells significantly increased, while that of Bcl-2 remarkably decreased following irradiation, whereas compound 12 significantly reduced the level of Bax and increased that of Bcl-2 in irradiated cells (*p* < 0.01). In conclusion, our findings indicate that compound 12 could inhibit radiation-induced apoptosis by modulating the p53 pathway.

### 3.10. Compound 12 Reduced IR-Induced G2/M Arrest by Downregulating the Expressions of p53 and p21

**Figure 8:**
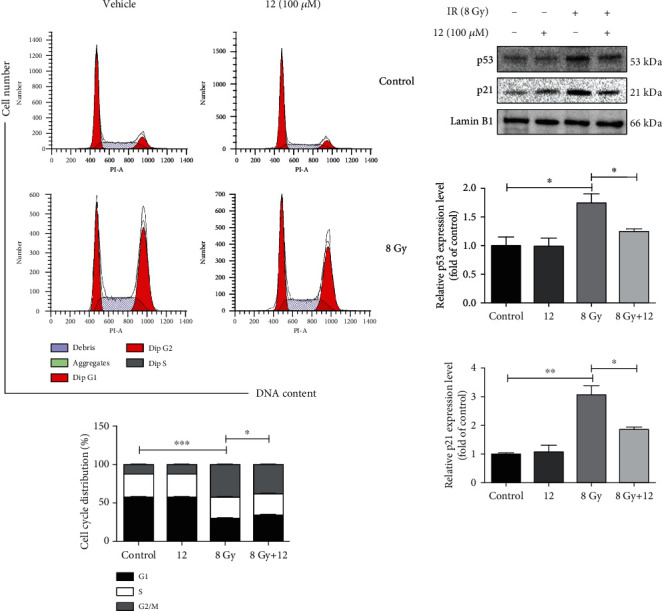
Compound 12 reduced IR-induced G2/M arrest and regulated the expression of p53 and p21. (a) The cell cycle distribution of IEC-6 cells was determined via flow cytometry at 12 h postirradiation. ^∗^*p* < 0.05, ^∗∗∗^*p* < 0.001; *n* = 3. (b) Analysis of the expression of p53 and p21 in IEC-6 cells. ^∗^*p* < 0.05, ^∗∗^*p* < 0.01; *n* = 3. The cells were cultured for 12 h after irradiation.

Since we observed that the expression of p53 in mouse small intestines could be downregulated by compound 12, we further investigated whether compound 12 can affect the cell cycle distribution of irradiated cells by regulating the expression of p53. We first analyzed the cell cycle distribution of IEC-6 cells using flow cytometry. As illustrated in Figure 8(a), IR induced G2/M arrest in IEC-6 cells at 12 h postirradiation (*p* < 0.001), and compound 12 effectively reduced G2/M arrest (*p* < 0.05). As reported in previous studies, p53 and p21 are required for sustaining G2/M arrest after IR-induced DNA injury [37]. Hence, we measured the expression levels of p53 and p21 in IEC-6 cells via Western blotting. As displayed in Figure 8(b), the expression levels of p53 and p21 in the nuclei of the cells evidently increased at 12 h postirradiation, whereas compound 12 remarkably decreased their expression levels. These findings indicate that compound 12 may alleviate IR-induced G2/M arrest by regulating the expression levels of p53 and p21.

### 3.11. Compound 12 Decreased IR-Induced Oxidative Stress by Regulating the Expression of Nrf2, NQO1, and NOX1

**Figure 9:**
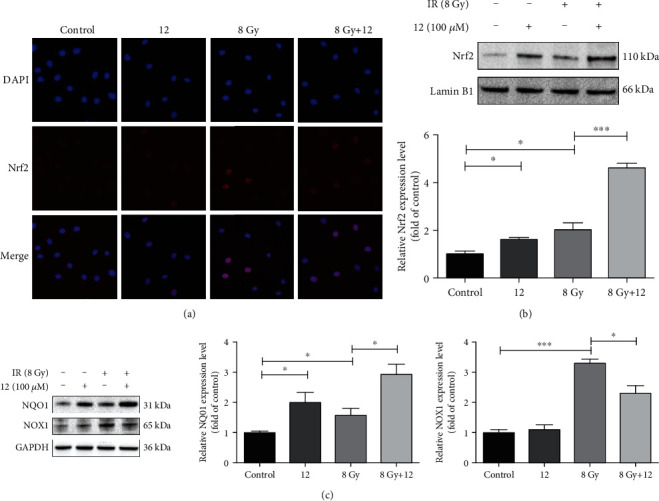
Compound 12 modulated the expression of Nrf2, NQO1, and NOX1 in IEC-6 cells. (a) Immunofluorescence images exhibiting the expression of Nrf2 (red: Nrf2; blue: DAPI). (b) Analysis of the expression of Nrf2 in the nuclei of the cells via Western blotting. (c) Analysis of the expression of NQO1 and NOX1 in the cytoplasm of the cells via Western blotting. The cells were cultured for 24 h after irradiation, and data are presented as the mean ± SEM (*n* = 3). ^∗^*p* < 0.05, ^∗∗∗^*p* < 0.001.

When activated under stress, the transcription factor of Nrf2 can enter the nucleus and promote the expression of antioxidant proteins, thereby regulating redox homeostasis in cells [38]. We first analyzed the expression of Nrf2 in IEC-6 cells via immunofluorescence staining. As illustrated in Figure 9(a), ionizing radiation promoted the nuclear translocation of Nrf2, and compound 12 further promoted this process. Next, we detected the expression level of Nrf2 in the cells via Western blotting. As demonstrated in Figure 9(b), the expression level of Nrf2 in the nuclei of irradiated cells increased significantly, and compound 12 further enhanced its expression (*p* < 0.05). In addition, treatment with compound 12 alone could also increase the level of Nrf2 (*p* < 0.05).

NQO1 is an antioxidant enzyme that can be upregulated by Nrf2 [39]. We assessed the expression level of NQO1 in the cytoplasm of IEC-6 cells using Western blotting. As displayed in Figure 9(c), the expression level of NQO1 in the IR group increased markedly compared with the control group (*p* < 0.05). Furthermore, the expression of NQO1 in the IR + 12 group increased significantly compared with the IR group (*p* < 0.05). NADPH oxidase (NOX) is one of the most important sources of ROS in the body, while NOX1 is a ubiquitous NADPH oxidase in epithelial cells [40]. We also evaluated the expression levels of NOX1 via Western blotting. As shown in Figure 9(c), IR increased the expression level of NOX1 (*p* < 0.001), while compound 12 reduced its expression (*p* < 0.05). These data indicate that compound 12 can decrease oxidative stress damage by upregulating the expression of Nrf2 and NQO1 and downregulating the expression of NOX1 in irradiated cells.

## 4. Discussion

In the current study, we synthesized a novel aminothiol compound 12 as a radioprotector. Since there is a higher number of thiol groups in this molecule compared to other radioprotectors, it can exert higher ROS scavenging and radioprotective activities. In addition, the thiols in the molecule are located away from the alkylamino backbone, thus eliminating ROS before they attack the DNA frame structure. Moreover, the amine and thiol groups in the molecule are capped, so its cell permeability and oral bioavailability can be increased compared with the compound previously reported by our laboratory [17]. We speculate that the capped group in the alkyl side chain of compound 12 could be cleaved by hydrolytic enzymes inside cells. As a result, it showed better radioprotective activities both *in vitro* and *in vivo* than the uncapped compound.

Bone marrow hematopoietic tissue is the most radiosensitive tissue in the body [41]. Myelosuppression and myeloid skewing are typical symptoms of radiation-induced hematopoietic injury [22, 23]. We showed that the oral administration of compound 12 could improve the survival rate of mice that received a lethal dose of TBI and alleviated myelosuppression and myeloid skewing induced by irradiation. Moreover, IR may deplete HSPCs. Here, we found that compound 12 markedly increased the frequencies of HSCs and HPCs and reduced the ROS levels in these cells. These results indicate that compound 12 can effectively prevent hematopoietic system damage and decrease oxidative stress induced by ionizing radiation.

Radiation therapy for malignant tumors in the abdomen can damage normal tissues, and small intestine is especially sensitive to ionizing radiation [42, 43]. We built an ABI model and observed that compound 12 could prevent structural damage to the small intestines caused by IR. The intestinal epithelium can continuously renew itself through ISCs at the bottom of intestinal crypts [27]. Our results showed that compound 12 could increase the survival of Lgr5^+^ ISCs, Paneth cells, and Ki67^+^ cells in the small intestines of IR-exposed mice. Overall, these results indicate that compound 12 can protect against radiation-induced intestinal injury by maintaining intestinal homeostasis and improving the regenerative capacity of ISCs.

**Figure 10:**
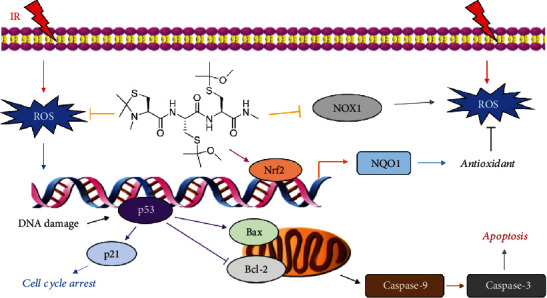
Proposed mechanisms underlying the radioprotective effect of compound 12. IR induces the generation of ROS, thereby causing DNA strand breaks, which can activate p53, promote apoptosis, and maintain cell cycle arrest. Compound 12 can decrease oxidative stress by eliminating ROS directly and regulating the expression levels of Nrf2, NQO1, and NOX1, thus inhibiting DNA damage, suppressing p53-dependent apoptosis, and relieving cell cycle arrest.

Ionizing radiation can increase the levels of ROS in irradiated cells, thus inducing DNA injuries, of which DSB is the most lethal. And *γ*-H2AX is an early marker of DSBs in the body. We found that compound 12 significantly decreased the expression of *γ*-H2AX, which suggested that it might reduce radiation-induced DSBs by eliminating ROS in irradiated cells. DNA strand breaks can induce the upregulation of p53 expression, thereby causing cell cycle arrest and promoting DNA repair or leading to cell apoptosis [44, 45]. Studies have shown that p53 plays an important role in both G1 and G2/M arrest, and p53's activation of p21 is essential for maintaining G2/M arrest induced by DNA damage [37]. Our results showed that IR could cause G2/M phase arrest in cells, and compound 12 effectively alleviated G2/M arrest; compound 12 could also downregulate the expressions of p53 and p21 in irradiated cells. These results indicate that compound 12 may reduce DNA damage by scavenging ROS in irradiated cells, thereby alleviating IR-induced cell cycle arrest (Figure 10).

Mitochondria play an important role in apoptosis. Studies have shown that p53 can change the permeability of the mitochondrial outer membrane by regulating the expression of the Bcl-2 protein family members (such as Bax, Bak, Bcl-2, and Bcl-X_L_), thus inducing the release of cytochrome c (Cyt c) [46, 47]. Cyt c can combine with Apaf-1 and activate the initiator caspase-9, which further activates the effectors caspase-3 and caspase-7, thereby inducing apoptosis [30, 48]. We showed that the expression levels of p53, Bax, caspase-9, and caspase-3 could be downregulated and that of Bcl-2 could be upregulated by compound 12. These results indicate that compound 12 may alleviate DNA strand breaks caused by excessive ROS, thus inhibiting p53-dependent apoptosis (Figure 10).

We believe that compound 12 can directly eliminate ROS in cells. In addition, we also investigated whether compound 12 could alleviate oxidative injury in radiated cells through other pathways. Nrf2 is an important transcription factor that can adjust redox homeostasis [38]. When activated by ROS or other factors, it can dissociate from Kelch-like ECH-associated protein 1 (Keap1) and enter the nucleus, thus inducing the expression of antioxidant proteins such as NQO1 [39]. We found that compound 12 could upregulate the levels of Nrf2 and NQO1 in IEC-6 cells, suggesting that it presumably targets Nrf2 or its upstream signaling molecules. Furthermore, Nrf2 can promote the repair of DSBs through mechanisms other than antioxidation [49]. It has been reported that Nrf2 could act as an ATR activator in the modulation of the cellular response to DNA damage [50]. Hence, we speculated that compound 12 could also alleviate DNA damage by activating ATR through the regulation of Nrf2. NOXs are key enzymes for redox signals and the main sources of ROS in the body, and NOX1 is a ubiquitous NOX in epithelial cells [40]. We found that compound 12 downregulated the expression level of NOX1 in irradiated cells. Collectively, these findings suggest that compound 12 could also mitigate IR-induced oxidative damage by regulating the expression of Nrf2, NQO1, and NOX1 in cells (Figure 10).

## 5. Conclusion

In summary, a new aminothiol compound 12 was designed and synthesized in this study. It could be administered orally to mice and displayed potent radioprotective activity. Our results demonstrated that compound 12 could improve the survival rates of mice that received fatal doses of irradiation. We showed that compound 12 effectively prevented TBI-induced hematopoietic damage and ABI-induced intestinal damage in mice. Furthermore, we proved that compound 12 could reduce oxidative stress and inhibit DNA damage, thereby alleviating IR-induced cell cycle arrest. Our experimental results also suggested that compound 12 might suppress radiation-induced apoptosis through the p53 pathway. Taken together, compound 12 can be a potential radioprotective agent, and the precise mechanisms underlying its radioprotective activities warrant further investigation in the future.

## Data Availability

The data used to support the findings of this study are included within the article.

## Supplementary Materials

Supplementary Materials There are 16 figures in the supplementary materials: the data for cell toxicity and radioprotective activity of compound 12 are shown in Figures S1 and S2; the data for the survival analysis of mice receiving compound 12 by gavage 0.5 h postirradiation is shown in Figure S3; the data for the characterizations of all compounds are shown in Figures S4–S16.
