# Supplementary material for: Design, Synthesis, and Biological Evaluation of a Novel Aminothiol Compound as Potential Radioprotector
Source: Oxid Med Cell Longev. 2021 Aug 21;2021:4714649. doi: 10.1155/2021/4714649 (PMC8405339; doi:10.1155/2021/4714649)
Supplement: Supplementary Materials — There are 16 figures in the supplementary materials: the data for cell toxicity and radioprotective activity of compound 12 are shown in Figures S1 and S2; the data for the survival analysis of mice receiving compound 12 by gavage 0.5 h postirradiation is shown in Figure S3; the data for the characterizations of all compounds are shown in Figures S4–S16. [file 4714649.f1.docx]

**Supplementary Materials**

**Design, Synthesis, and Biological Evaluation of** **a Novel Aminothiol Compound as Potential Radioprotector**

Xuejiao Li, Xinxin Wang, Longfei Miao, Yuying Guo, Renbin Yuan, Jingming Ren, Yichi Huang, Hongqi Tian

Tianjin Key Laboratory of Radiation Medicine and Molecular Nuclear Medicine, Institute of Radiation Medicine, Peking Union Medical College and Chinese Academy of Medical Science, Tianjin 300192, China

Correspondence should be addressed to Hongqi Tian; tianhongqi@irm-cams.ac.cn

**Experimental Section**

**Materials and methods**

**Cell viability assay**

For the cytotoxicity experiment, IEC-6 cells were plated in 96**-**well plates (5 × 10^3^ cells per well), incubated overnight, and treated with compound **12** at concentrations from 0 to 1 mmol/L. After 24 h, the cells were washed once, and cell viability was measured using the luminescence-based CellTiter Glo TM assay according to the manufacturer’s protocols (Promega, Madison, WI, USA).

To evaluate the radioprotective effect of compound **12**, IEC-6 cells were plated in 96**-**well plates (5 × 10^3^ cells per well) and incubated overnight. The cells were pretreated with compound **12** 2 h before γ-radiation (6.0, 8.0, 10.0 Gy). After 24 h, cell viability was measured as described above.

**Acute toxicity assay**

A total of 40 mice were randomly assigned to four groups (*n* = 10). Each group received a different dose of compound **12** (0, 800, 1200, and 1600 mg/kg) by oral gavage. The toxicity of the treatments and the death of mice were monitored and recorded for the next 14 days.

**Survival study**

The mice were randomly divided into 3 groups (*n* = 10 per group), namely control, TBI + vehicle, and TBI + **12** (1200 mg/kg). Mice in the irradiation groups received 8.0 Gy TBI. The mice of the TBI + **12** group were treated with compound **12** at the indicated dose 0.5 h after radiation. The survival status of each mouse was observed for 30 days after irradiation, and the results were expressed as survival rates.

**Results**

**Cytotoxicity and** **radioprotective activity of compound 12**

Initially, we evaluated the toxicity of compound **12** on IEC-6 cells. As illustrated in Figure S1, compound **12** had no toxic effect on IEC-6 cells at lower concentrations and exhibited more pronounced inhibitory effect on the cells at concentrations higher than 300 μmol/L (*p* < 0.05). Thus, a concentration 100 μmol/L was used for *in vitro* experiments.

Then we investigated the radioprotective activity of compound **12** on IEC-6 cells (Figure S2). The survival rates of IEC-6 cells decreased significantly after 8.0 Gy or 10.0 Gy irradiation (*p* < 0.05). For the groups pretreated with compound **12**, the cell survival rates increased markedly compared with the cells treated with 8.0 Gy or 10.0 Gy irradiation only (*p* < 0.05), indicating that compound **12** has radioprotective activity on IEC-6 cells at the concentration of 100 μmol/L.

**Acute toxicity of compound 12**

During the acute toxicity test, there was no death in all groups. Nevertheless, hypoactivity was observed in three mice at a dose of 1600 mg/kg after administration. Therefore, three doses below 1600mg/kg were selected in the survival study.

**Compound 12 did not improve the survival of TBI-exposed mice**

As illustrated in Figure S3, all mice in the TBI + vehicle group died within 14 days, but no death was observed in the control group. Besides, all mice in the TBI + **12** group died within 19 days, so compound **12** did not significantly increase the survival rate of mice when administrated 0.5 h after irradiation.

**Synthetic procedures Section**

**Synthesis of all intermediates and the target compound**

**S-trityl-L-cysteine** (**2**). L-cysteine•HCl (10.00 g, 63.45 mmol) was dissolved in DMF (120.ml) followed by addition of TrtCl (19.46 g, 69.79 mmol). The solution was heated at 60-65 °C for 8h, after cooled to room temperature, 10% NaOAc (300 ml) aqueous solution was added under stirring. White solid was precipitated, the mixture was filtered and the residue was washed with water (300 ml) and acetone (200 ml), after dried (Na_2_SO_4_), S-trityl-L-cysteine was obtained (yield: 76.1%). ^1^H NMR (400 MHz, DMSO-d6) δ 7.28 (m, 18H), 2.92(dd, 1H), 2.59(dd, 1H), 2.41(dd, 1H).

**N-(tert-butoxycarbonyl)-S-trityl-L-cysteine (3).** S-trityl-L-cysteine (5.00 g, 13.76 mmol) was dissolved in the mixture of dioxane (40 ml), NaOH (14 ml, 1.00 M) and water (20 ml). The solution was stirred at ice bath and Boc-anhydride was added, the mixture was stirred at room temperature for 8 h and concentrated in vacuo to 20-25 ml, diluted with EtOAc (ethyl acetate) (100 ml) and adjust PH to 2-3 with NaHSO_4_ aqueous at ice bath. Then solution was extracted with EtOAc (100 ml×3). The organic phase was washed with water (100 ml) and dried with Na_2_SO_4_, concentrated in vacuo and purified by silica gel chromatography (DCM/Methanol (v/v = 5/1)) to give N-(tert-butoxycarbonyl)-S-trityl-L-cysteine as white solid (yield: 86.2%). ^1^H NMR (400MHz, CDCl_3_) *δ* 7.44-7.41(m, 6H), 7.32-7.28(m, 7H), 7.26-7.21(M, 2H), 4.92(d, 1H, *J* = 8.0 Hz), 4.13(d, 1H, *J* = 8.0 Hz), 2.70(d, 2H, *J* = 8.0 Hz), 1.46(s, 9H).

**N-(tert-butoxycarbonyl)-N-methyl-S-trityl-L-cysteine (4).** NaH (436.00 mg, 10.90 mmol) was suspended in THF (14.00 ml) and slow added of N-(tert-butoxycarbonyl)-S-trityl-L-cysteine (2.10 g, 4.53 mmol) with THF (6 ml) at ice bath followed by addition of CH_3_-I (0.93 ml, 14.95 mmol), the solution was stirred overnight at room temperature. The mixture was quenched by phosphate buffer (PH=7), saturated aqueous NH_4_Cl solution was added to pH 2-3, and then extracted with EtOAc (50 ml). The organic phase was washed with water (50 ml) and dried with Na_2_SO_4_, concentrated in vacuo, and purified by silica gel chromatography (DCM/Methanol (v/v = 5/1)) to give N-(tert-butoxycarbonyl)-N-methyl-S-trityl-L-cysteine as white solid (yield: 60.1%). ^1^H NMR (400MHz, D_2_O) *δ* 7.37-7.25(m, 15H), 3.97(s, 1H), 2.67(s, 2H), 2.61(t, 3H), 1.39(S, 4H,), 1.28(s, 5H).

**(9H-fluoren-9-yl) methyl (R)-(1-(methylamino)-1-oxo-3-(tritylthio) propan-2-yl) carbamate (6).** N-(((9H-fluoren-9-yl) methoxy) carbonyl)-S-trityl-L-cysteine (10.00 g, 17.07 mmol) was dissolved in THF (50 ml) at 0-5 ℃ followed by addition of CDI (5.59 g, 38.48 mmol). The solution was stirred at 0-5 ℃ for 2 h in N_2_ atmosphere, aqueous CH_3_NH_2_ solution (3.03 ml, 68.28 mmol) was added stirred at 0-5 ℃ for 2 h in N_2_ atmosphere. The mixture was quenched by 2M HCl (60 ml) and extraction by DCM (200 ml). The organic phase was washed with saturated NaCl aqueous solution (300 ml) and dried with Na_2_SO_4_, concentrated in vacuo*,* methanol (20 ml) was added in crude product and stirred for overnight at room temperature. White solid was precipitated, the mixture was filtered to give (9H-fluoren-9-yl) methyl (R)-(1-(methylamino)-1-oxo-3-(tritylthio) propan-2-yl) carbamate. The filtrate was concentrated in vacuo and purified by silica gel chromatography (DCM/Methanol (v/v = 5/1)) to give (9H-fluoren-9-yl)methyl (R)-(1-(methylamino)-1-oxo-3-(tritylthio)propan-2-yl)carbamate as white solid (yield: 92.3%). ^1^H NMR (400MHz, D_2_O) *δ* 7.89(d, 2H, *J* = 8.0 Hz), 7.81(d, 1H, *J* = 4.0 Hz), 7.74(d, 2H, *J* = 8.0 Hz), 7.66(d, 1H, *J* = 8.0 Hz), 7.41(t, 2H), 7.35-7.24(m, 15H), 4.32-4.20(m, 3H), 4.00(d, 1H, *J* = 8.0 Hz), 2.53(d, 3H, *J* = 4.0 Hz), 2.39(d, 2H, *J* = 8.0 Hz).

**(R)-2-amino-N-methyl-3-(tritylthio) propanamide (7).** (9H-fluoren-9-yl) methyl (R)-(1-(methylamino)-1-oxo-3-(tritylthio) propan-2-yl) carbamate (2.00 g, 3.34 mmol) was dissolved in DMF (20 ml) followed by addition of piperidine (0.07 ml, 0.66 mmol). The solution was at stirred at room temperature for 4 h. After detected and the mixture was washed with saturated aqueous NaCl solution (50 ml), then extracted with DCM (100 ml×3) and dried with Na_2_SO_4_, concentrated in vacuo and purified by silica gel chromatography (DCM/Methanol (v/v = 5/1)) to give (R)-2-amino-N-methyl-3-(tritylthio) propanamide as slightly yellow white solid (yield: 69.7%). ^1^H NMR (400MHz, D_2_O) *δ* 7.77(d, 1H, *J* = 4.0 Hz), 7.36-7.23(m, 15H), 3.08(m, 1H), 2.55(d, 3H, *J* = 8.0 Hz), 2.39-2.35(m, 1H), 2.21-2.16(m, 1H), 1.80(s, 2H).

**(9H-fluoren-9-yl) methyl ((R)-1-(((R)-1-(methylamino)-1-oxo- 3-(tritylthio) propan-2-yl) amino)-1-oxo-3-(tritylthio) propan-2-yl) carbamate (8).** N-(((9H-fluoren-9-yl) methoxy) carbonyl)-S-trityl-L-cysteine (100.00 mg, 0.17 mmol) was dissolved in DCM (5 ml) and HOBt (34.50 mg, 0.25 mmol), EDCI (48.90 mg, 0.25 mmol) were added. The solution was stirred at room temperature for 5min and then (R)-2-amino-N-methyl-3-(tritylthio) propanamide (76.80 mg, 0.21 mmol) was added, After 30 min later, the mixture was washed with saturated aqueous NaCl solution (20 ml), then extracted with DCM (50 ml ×3) and dried with Na_2_SO_4_, concentrated in vacuo and purified by silica gel chromatography (DCM/Methanol (v/v = 5/1)) to give (9H-fluoren-9-yl)methyl ((R)-1-(((R)-1-(methylamino)-1-oxo-3-(tritylthio)propan-2-yl)amino)-1-oxo-3-(tritylthio)propan-2-yl)carbamate as white solid (yield: 99.6%). ^1^H NMR (400MHz, CDCl_3_) *δ* 7.76(m, 2H), 7.58(s, 2H), 7.45-7.16(m, 34H), 6.34(d, 1H, *J* = 8.0 Hz), 5.02(d, 1H, *J* = 8.0 Hz), 4.48-4.19(m. 4H), 3.84-3.78(m, 1H), 3.65(s, 3H), 2.69-2.59(m, 4H).

**(R)-2-amino-N-((R)-1- (methylamino) -1-oxo-3- (tritylthio) propan-2-yl)-3- (tritylthio) propanamide (9).** (9H-fluoren-9-yl) methyl ((R)-1-(((R)-1-(methylamino)-1-oxo-3-(tritylthio) propan-2-yl) amino)-1-oxo-3-(tritylthio) propan-2-yl) carbamate (3.60 g, 3.80 mmol) was dissolved in DMF (15 ml) and piperidine (0.07 ml, 0.76 mmol) was added. The solution was stirred at room temperature for 4 h, washed with saturated aqueous NaCl solution (30 ml), then extracted with DCM (50 ml×3) and dried with Na_2_SO_4_, concentrated in vacuo, and purified by silica gel chromatography (DCM/Methanol (v/v = 5/1)) to give (R)-2-amino-N-((R)-1-(methylamino)-1-oxo-3-(tritylthio)propan-2-yl)-3-(tritylthio)propanamide as white solid (yield: 47.4%). ^1^H NMR (400MHz, CDCl_3_) *δ* 7.45-7.19(m, 30H), 7.10(d, 1H, *J* = 8.0 Hz), 6.26(s, 1H), 4.02(d, 1H, *J* = 8.0 Hz), 2.82-2.69(m, 3H), 2.60(d, 3H, *J* = 4.0 Hz), 2.53-2.48(m, 2H).

**Tert-butyl methyl((4R,7R,10R)-3,6,9-trioxo-13,13,13-triphenyl- 4,7-bis((tritylthio) methyl)-12-thia-2,5,8-triazatridecan-10-yl) carbamate (10).** N-(tert-butoxycarbonyl)-N-methyl-S-trityl-L-cysteine (509.00 mg, 1.07 mmol) was dissolved in DCM (10 ml) and HOBt (218.00 mg, 1.61mmol), EDCI (309.00 mg, 1.61 mmol) were added. The solution was stirred at room temperature for 5min and then (R)-2-amino-N-((R)-1-(methylamino)-1-oxo-3-(tritylthio)propan-2-yl)-3-(tritylthio)propanamide (924.00 mg, 1.28 mmol) was added, after 30min later, the mixture was washed with saturated aqueous NaCl solution (30 ml), then extracted with DCM (50 ml ×3) and dried with Na_2_SO_4_, concentrated in vacuo and purified by silica gel chromatography (DCM/Methanol (v/v = 5/1)) to give tert-butyl methyl((4R,7R,10R)-3,6,9-trioxo-13,13,13-triphenyl-4,7-bis((tritylthio)methyl)-12-thia-2,5,8-triazatridecan-10-yl)carbamate as white solid (yield: 77.9%). ^1^H NMR (400MHz, CDCl_3_) *δ* 7.45-7.12(m, 45H), 6.57(t, 2H), 5.64(s, 1H), 4.18(d, 2H,), 3.63(d, 1H), 2.84-2.28(m, 12H), 1.42(s, 9H).

**(R)-3-mercapto-N-((R)-3-mercapto-1-(((R)-3-mercapto-1- (methylamino) -1- oxopropan-2-yl) amino) -1-oxopropan-2-yl)-2-(methylamino) propanamide** **trifluoroacetate (11).** Tert-butyl methyl((4R,7R,10R)-3,6,9-trioxo-13,13,13-triphenyl-4,7-bis((tritylthio)methyl)-12-thia-2,5,8-triazatridecan-10-yl)carbamate (985.00 mg, 0.83 mmol) was dissolved in mixture solution DCM:TFA:TIPS (v/v/v=50:47:3) (15 ml) The solution was stirred at room temperature for 5 min under N_2_ atmosphere, concentrated in vacuo and followed by addition of ether (45 ml) was stirred at ice bath. White solid was precipitated, filtered, and washed with ether (100 ml) to give (R)-3-mercapto-N-((R)-3-mercapto-1-(((R)-3-mercapto-1-(methylamino)-1-oxopropan-2-yl)amino)-1-oxopropan-2-yl)-2-(methylamino) propanamide trifluoroacetate (yield: 83.9%). ^1^H NMR (400MHz, DMSO-d6) *δ* 8.89 (d, 3H, *J* = 4.0 Hz), 8.38 (d, 1H, *J* = 8.0 Hz), 7.96 (d, 1H, *J* = 8.0 Hz), 4.53 (dd, 1H), 4.33 (dd, 1H), 4.04 (m, 1H), 3.38 (m, 1H), 2.91(m, 2H), 2.78-2.71(m, 3H), 2.61-2.56 (m, 3H), 2.50 (m, 3H), 2.29 (m, 1H), 1.28 (m, 1H), 1.29(m, 1H).

**(R)-2,2,3-trimethyl-N-((6R,9R)-3,3,12,12-tetramethyl-6-(methylcarbamoyl)-8-oxo-2,13-dioxa-4,11-dithia-7-azatetradecan-9-yl)thiazolidine-4-carboxamide (12).** (R)-3-mercapto-N-((R)-3-mercapto-1-(((R)-3-mercapto-1- (methylamino) -1- oxopropan-2-yl) amino) -1-oxopropan-2-yl)-2-(methylamino) propanamide trifluoroacetate (450 mg, 0.96 mmol) was added to a mixture of 2,2-dimethoxypropane (13.5 mL, 0.11 mol), acetone (4.5 mL, 0.06 mol) and montmorillonite (90 mg) under nitrogen atmosphere. And the solution was stirred at room temperature for 24 h. Then, the reaction mixture was filtered and evaporated to dryness. And the resulting residue was purified by neutral aluminum oxide column chromatography (DCM/Methanol (v/v = 350/1)) to give (R)-2,2,3-trimethyl-N-((6R,9R)-3,3,12,12-tetramethyl-6-(methylcarbamoyl)-8-oxo-2,13-dioxa-4,11-dithia-7-azatetradecan-9-yl)thiazolidine-4-carboxamide as white solid (yield: 12.5%). ^1^H NMR (400 MHz, CDCl_3_) δ 7.95 (d, *J* = 7.4 Hz, 1H), 7.36 (d, *J* = 8.0 Hz, 1H), 6.78 (d, *J* = 4.0 Hz, 1H), 4.68 (dd, *J* = 13.3, 5.4 Hz, 1H), 4.56 (dd, *J* = 13.6, 7.2 Hz, 1H), 3.57 (dd, *J* = 9.0, 5.2 Hz, 1H), 3.42 (dd, *J* = 11.7, 9.0 Hz, 1H), 3.31 (d, *J* = 1.8 Hz, 6H), 3.18 (dd, *J* = 13.8, 5.3 Hz, 1H), 3.10 (dd, *J* = 11.8, 5.2 Hz, 1H), 2.96 (dd, *J* = 13.0, 6.8 Hz, 3H), 2.84 (d, *J* = 4.8 Hz, 3H), 2.39 (s, 3H), 1.67 – 1.53 (m, 18H); ^13^C NMR (101 MHz, CDCl_3_) δ 173.44, 170.08, 170.02, 86.78, 86.43, 74.88, 72.07, 53.00, 52.90, 50.46, 50.31, 35.64, 32.63, 30.08, 29.86, 29.15, 28.19, 27.58, 26.22, 24.22; HRMS (ESI): *m/z* [M+Na]^+^ calcd for C_22_H_42_N_4_NaO_5_S_3_^+^: 561.2210, found: 561.2219.

**HPLC Method**

Compound **12** was chromatographed on a C18 column (250mm X 4.6mm, 5μm, Inertisl ODS-SP) with the column temperature of 25 ℃ and detected at 214 nm. The mobile phases were A: water (15%), B: acetonitrile (85%) with flow rate of 1.0 mL/min.

**Figures**


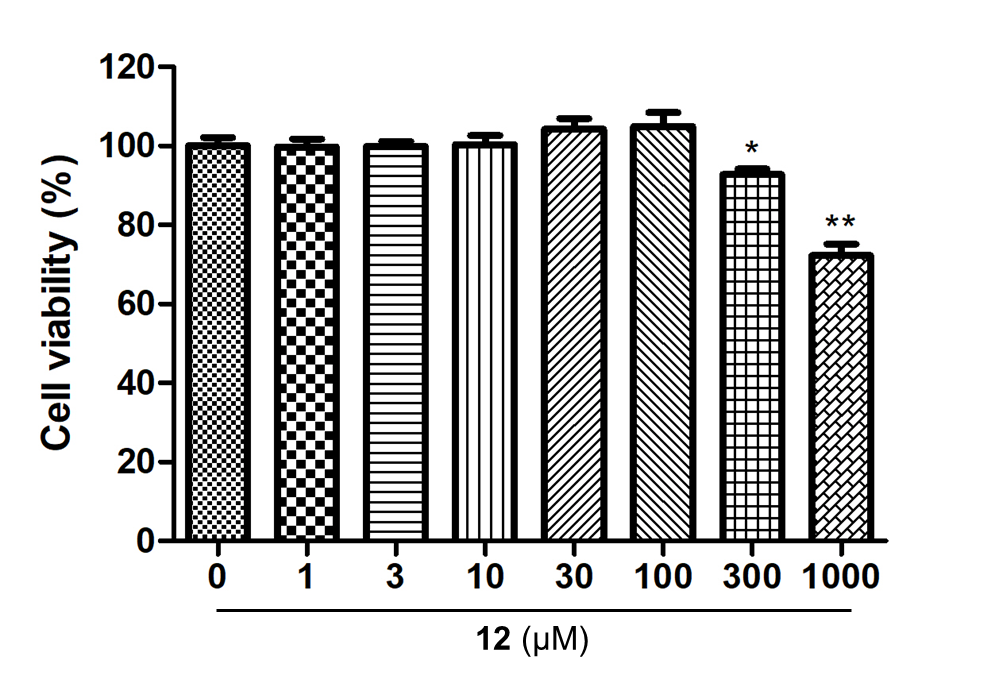


**Figure S1.** Cell toxicity of compound **12** on IEC-6 cells. Data are presented as the mean ± SEM (*n* = 3). **p* < 0.05 compared with the control group, ***p* < 0.01 compared with the control group.


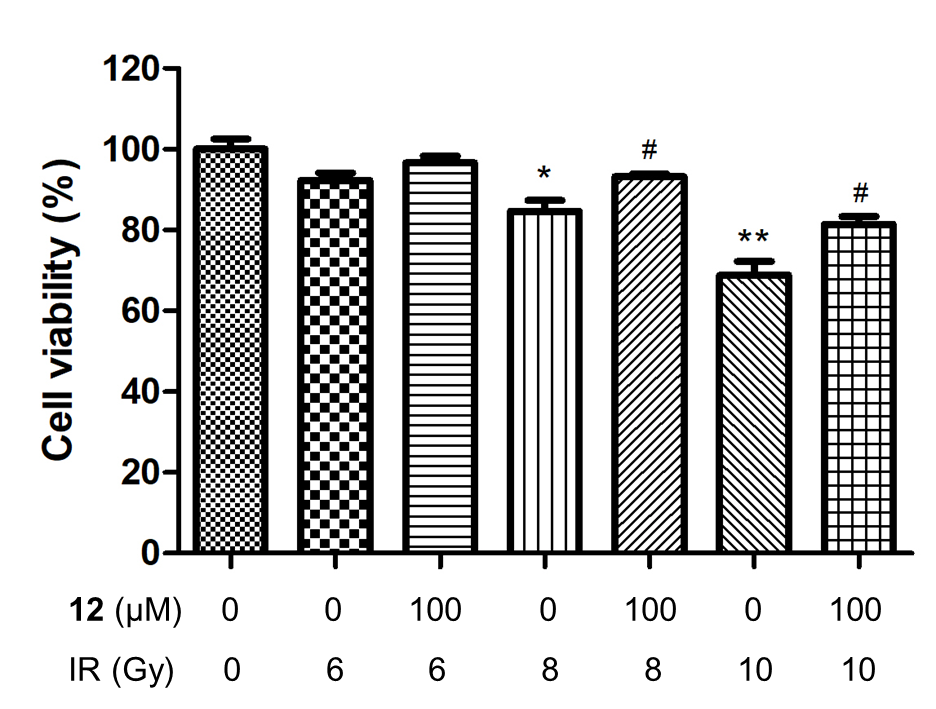


**Figure S2.** Radioprotective activity of compound **12** on IEC-6 cells. Data are presented as the mean ± SEM (*n* = 3). **p* < 0.05 compared with the control group, ***p* < 0.01 compared with the control group, ^#^*p* < 0.05 compared with the corresponding irradiation group.


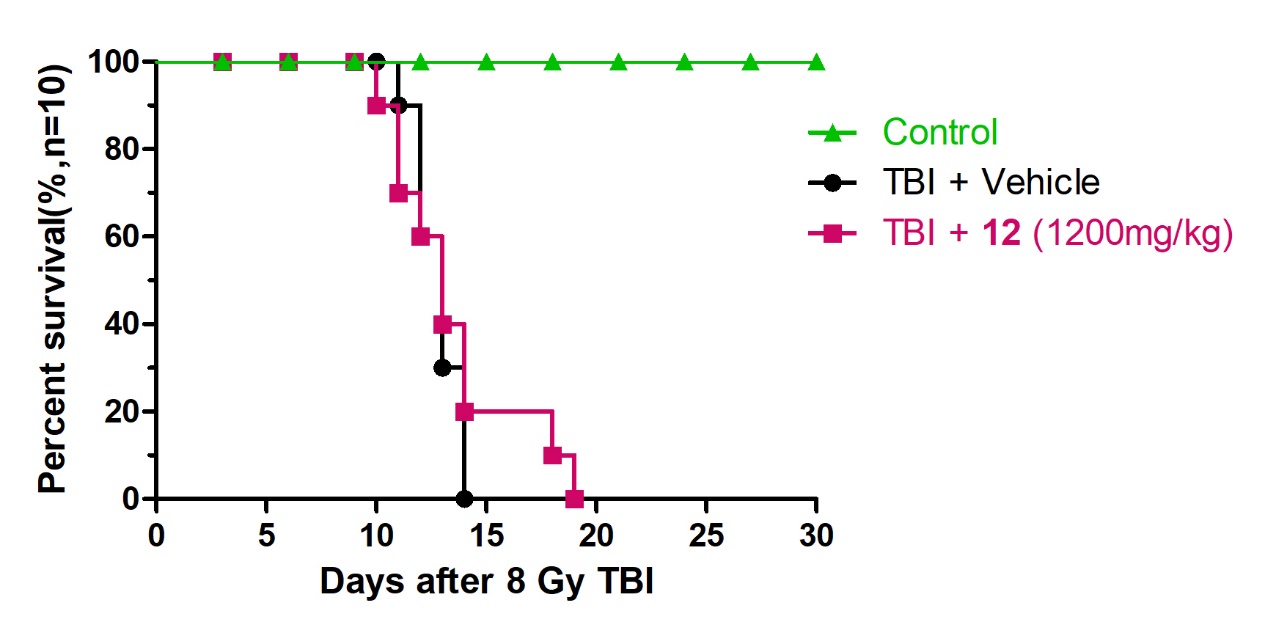


**Figure S3.** Survival analysis of mice exposed to 8.0 Gy TBI.

**Analysis spectra for** **all intermediates and the target compound**


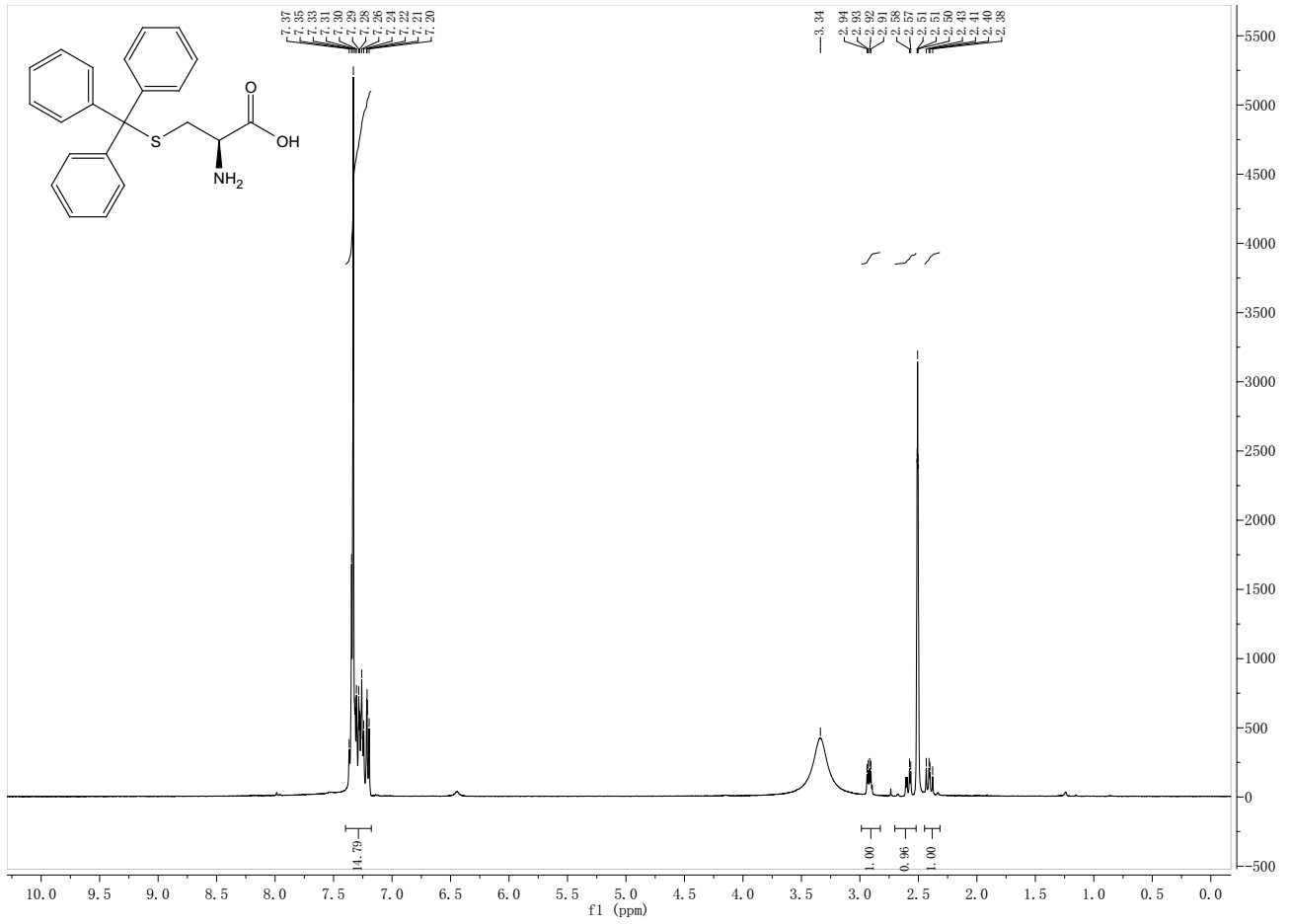


**Figure S4.** ^1^H NMR spectrum of compound **2**.


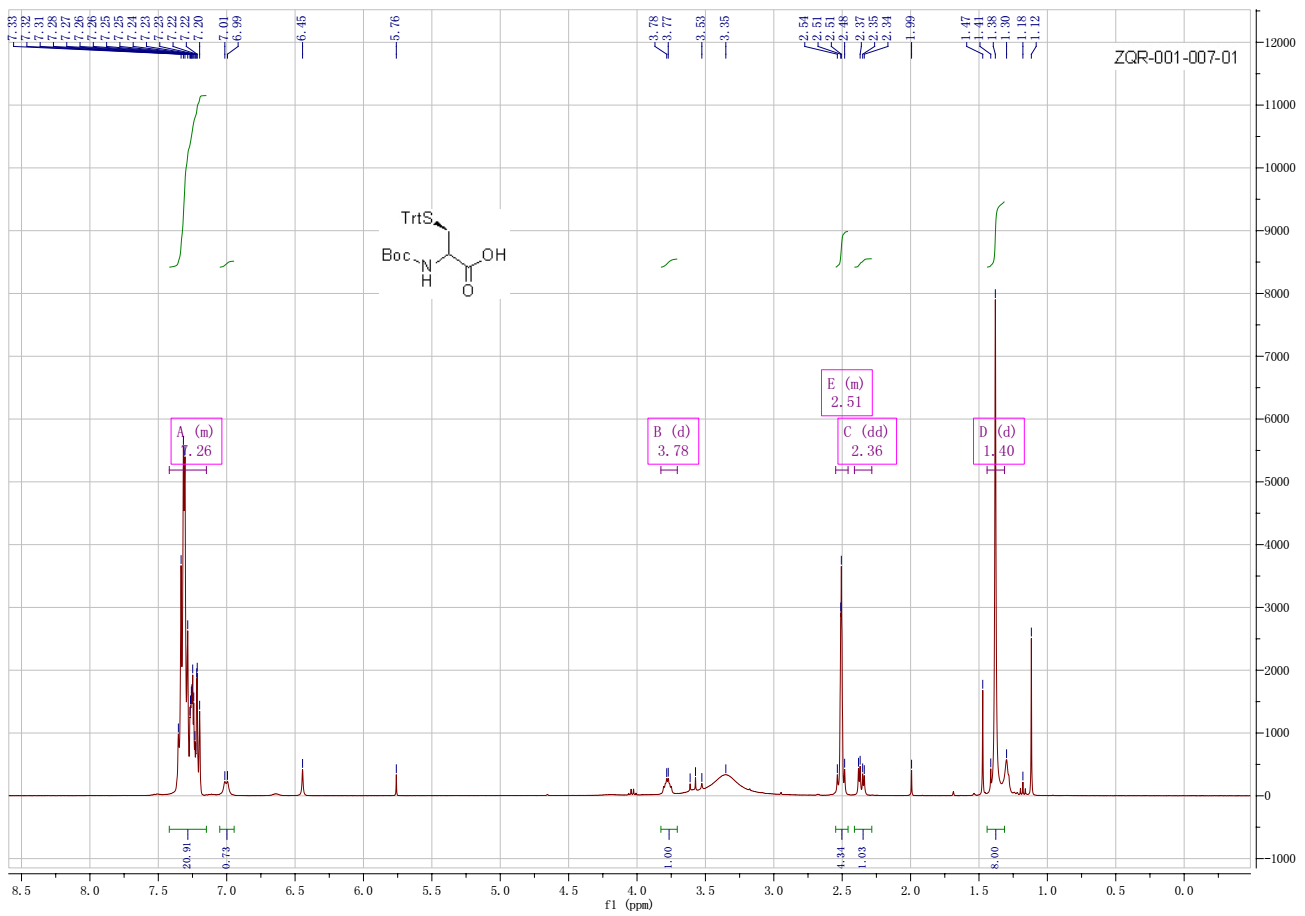


**Figure S5.** ^1^H NMR spectrum of compound **3**.


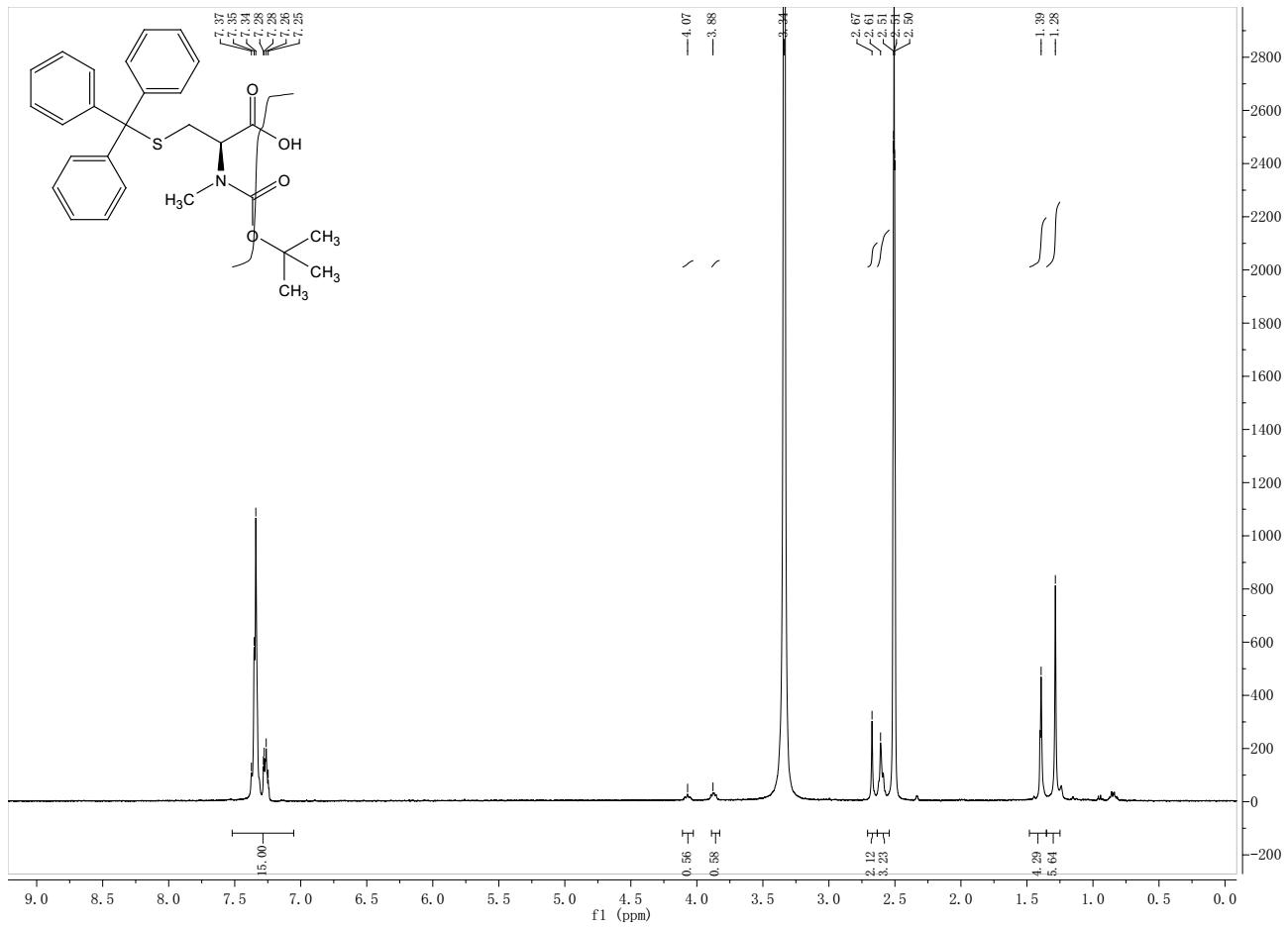


**Figure S6.** ^1^H NMR spectrum of compound **4**.


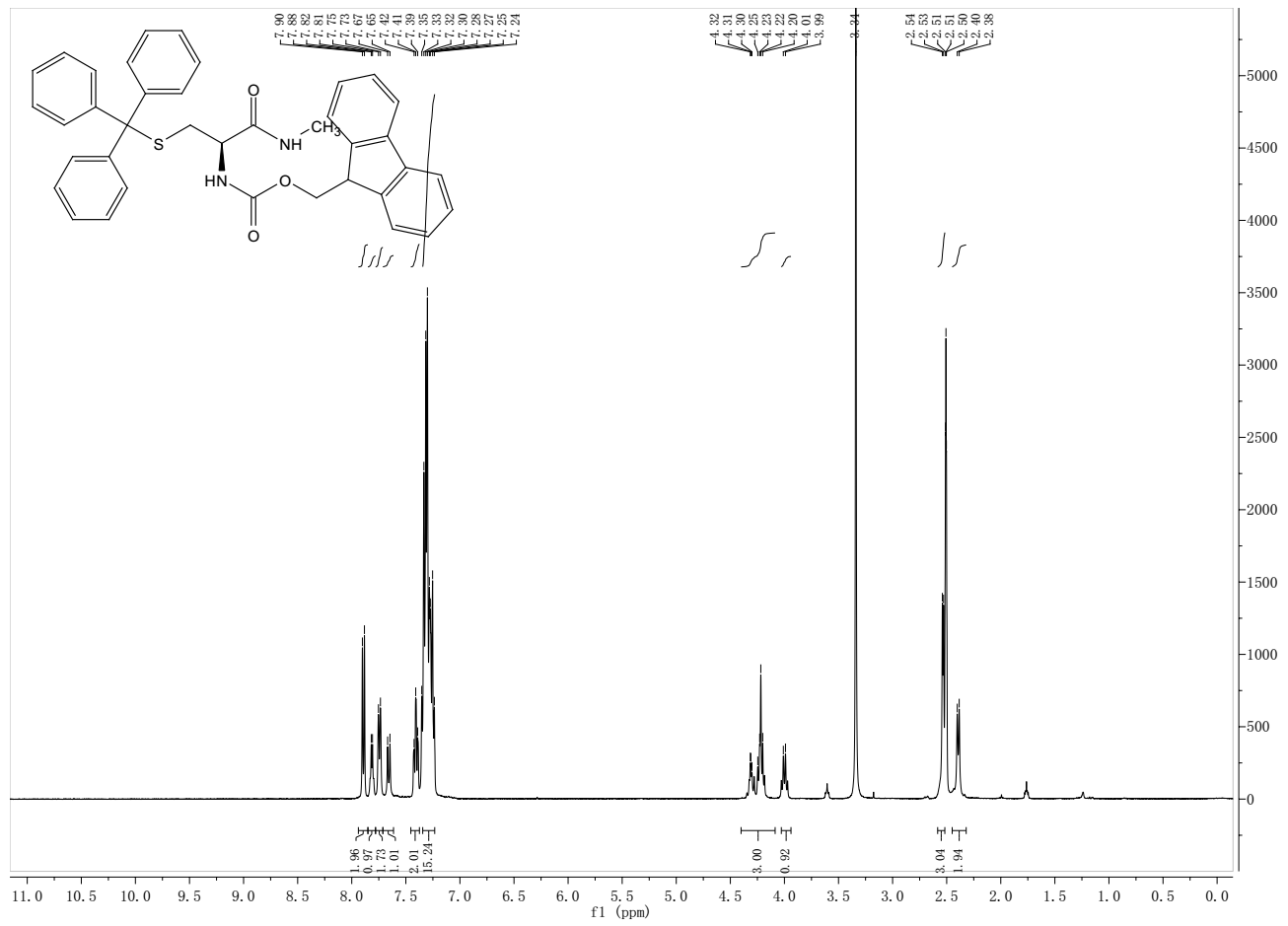


**Figure S7.** ^1^H NMR spectrum of compound **6**.


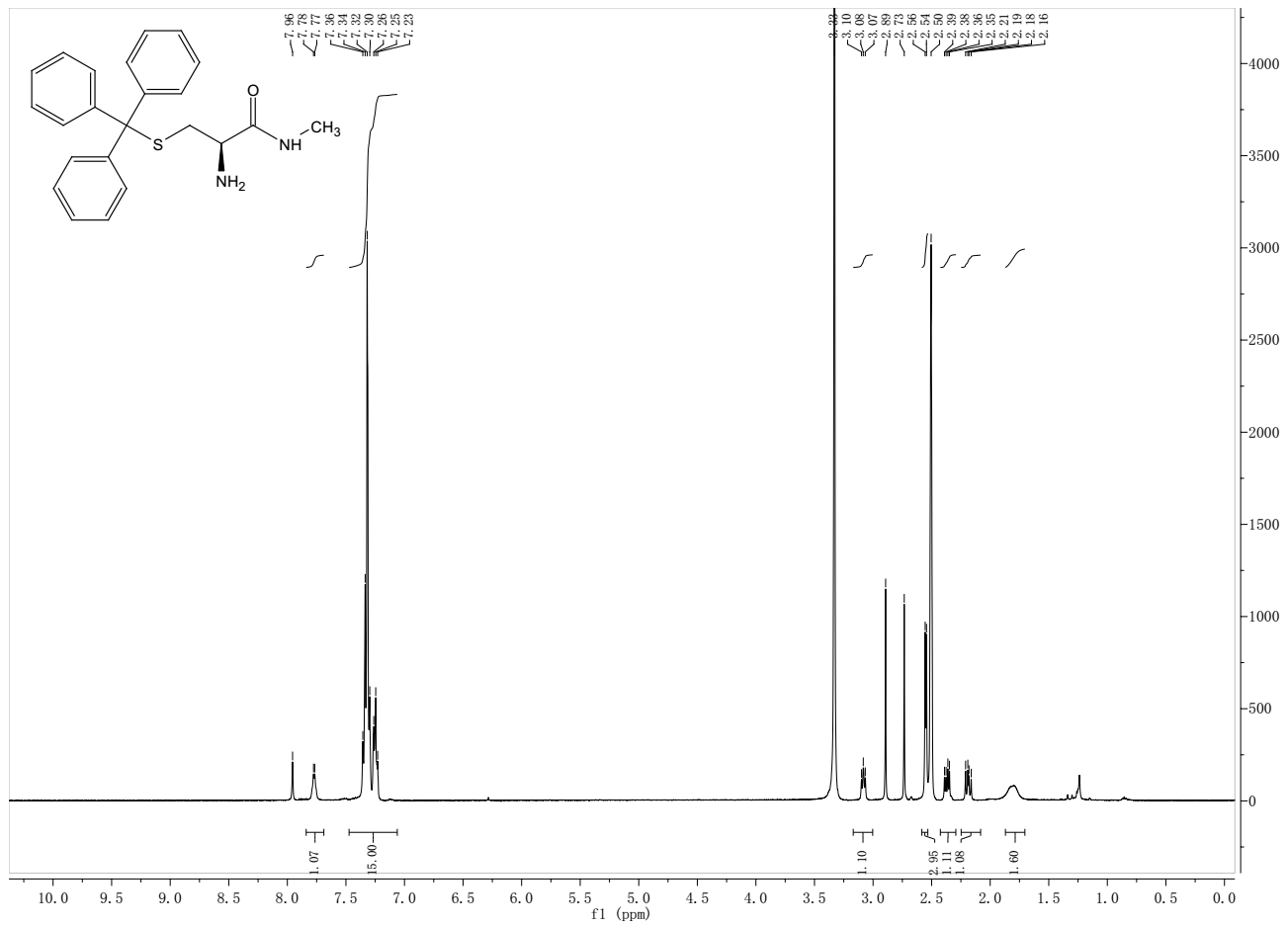


**Figure S8.** ^1^H NMR spectrum of compound **7**.


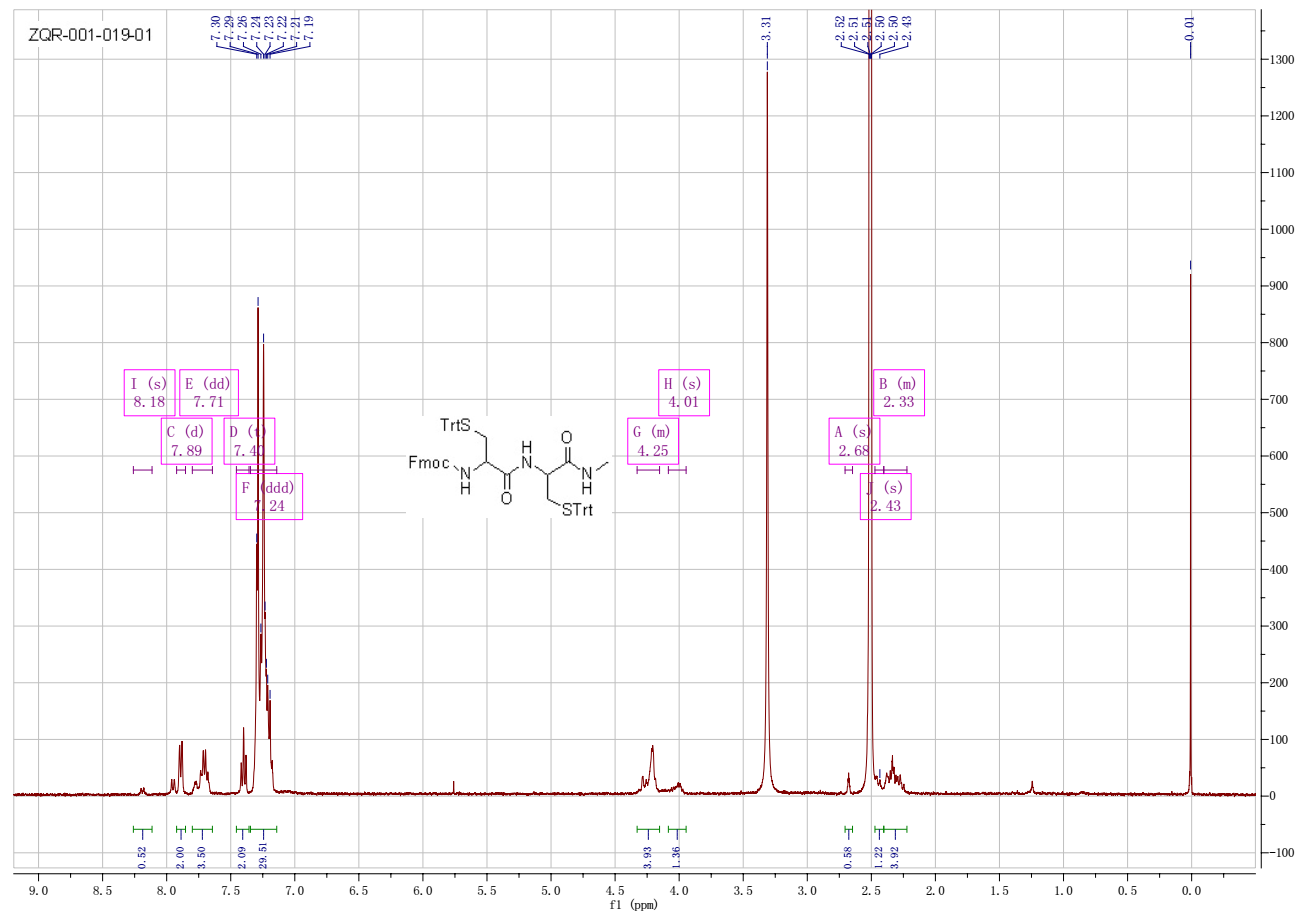


**Figure S9.** ^1^H NMR spectrum of compound **8**.


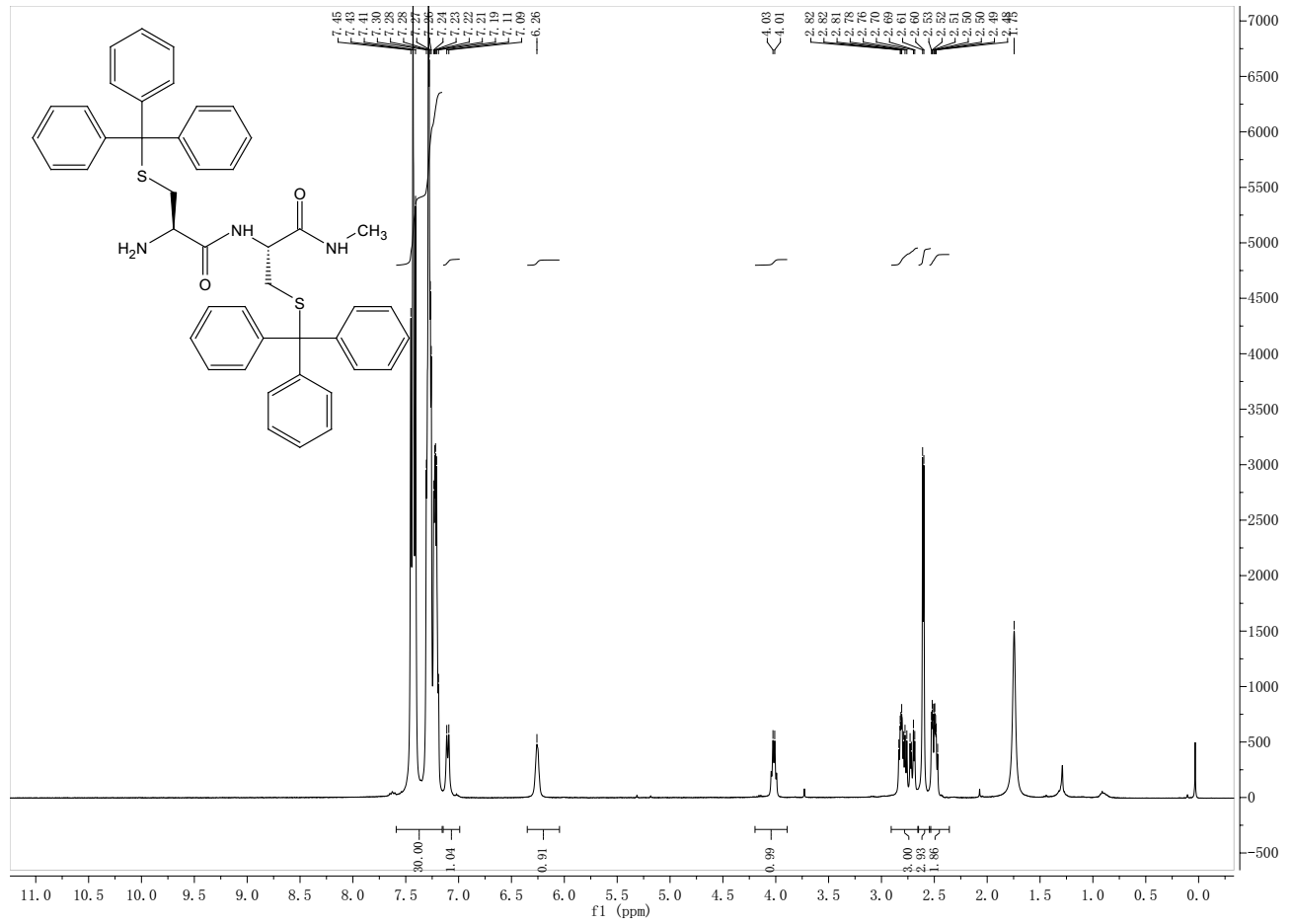


**Figure S10.** ^1^H NMR spectrum of compound **9**.


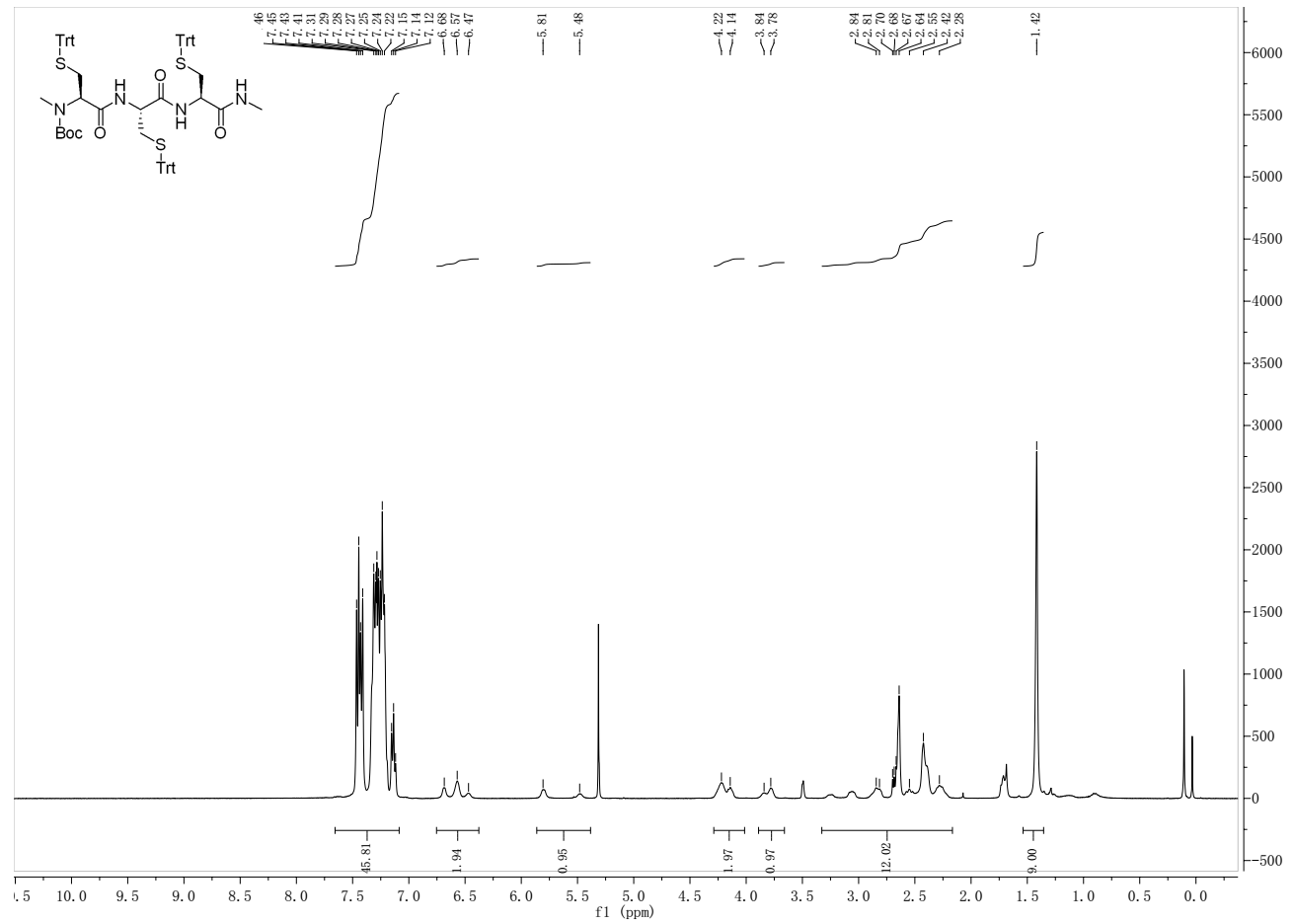


**Figure S11.** ^1^H NMR spectrum of compound **10**.


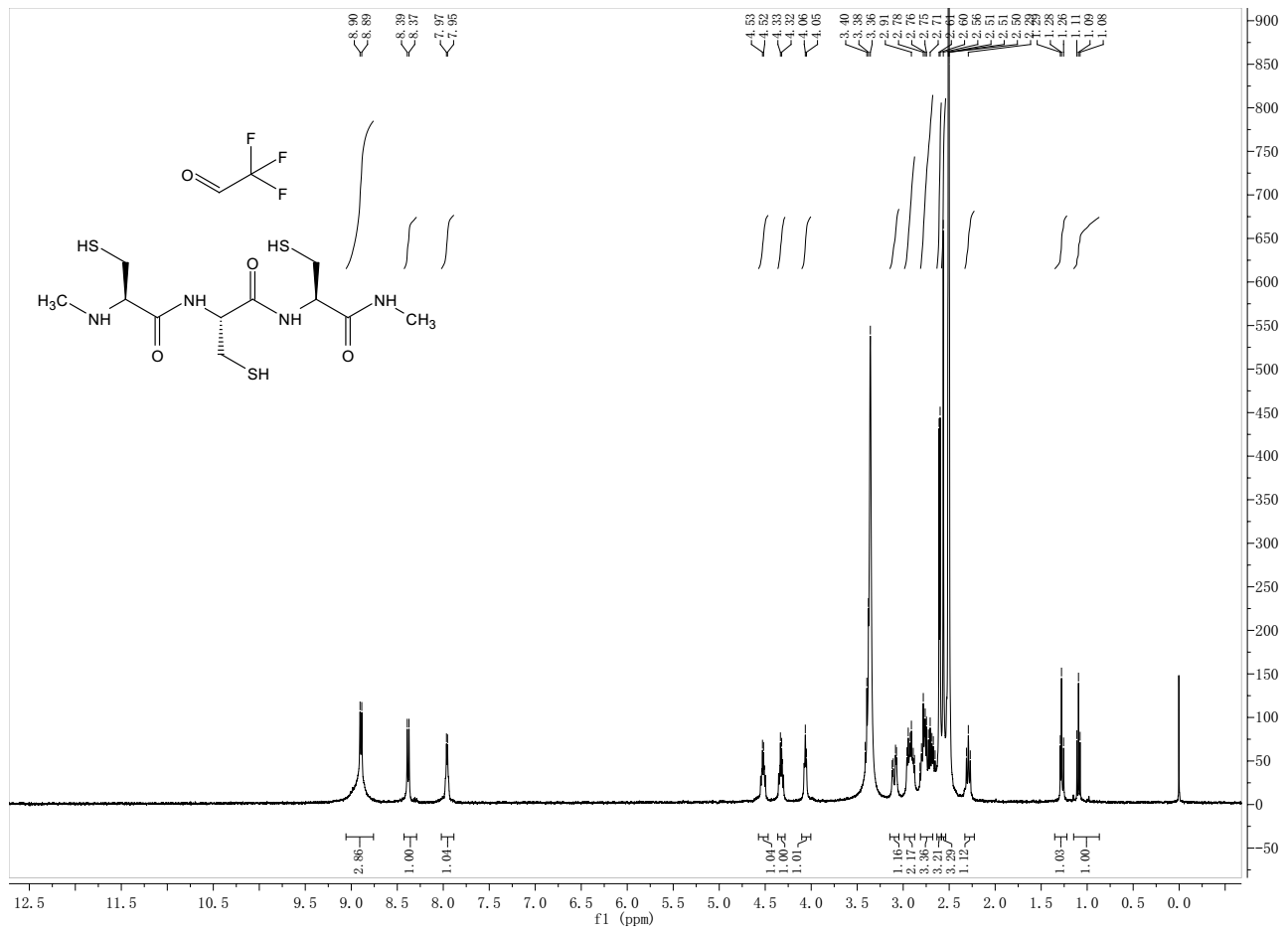


**Figure S12.** ^1^H NMR spectrum of compound **11**.


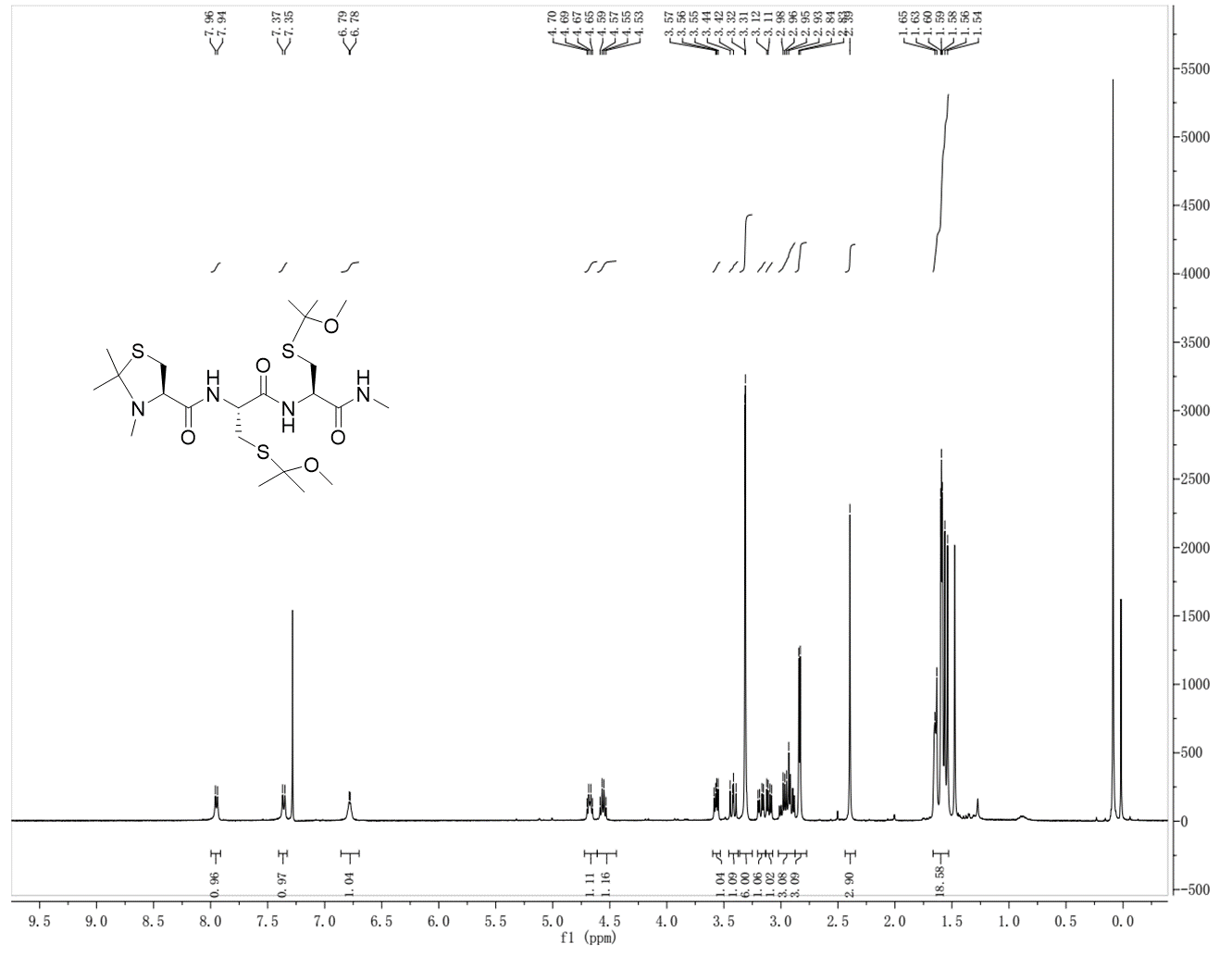


**Figure S13.** ^1^H NMR spectrum of compound **12**.


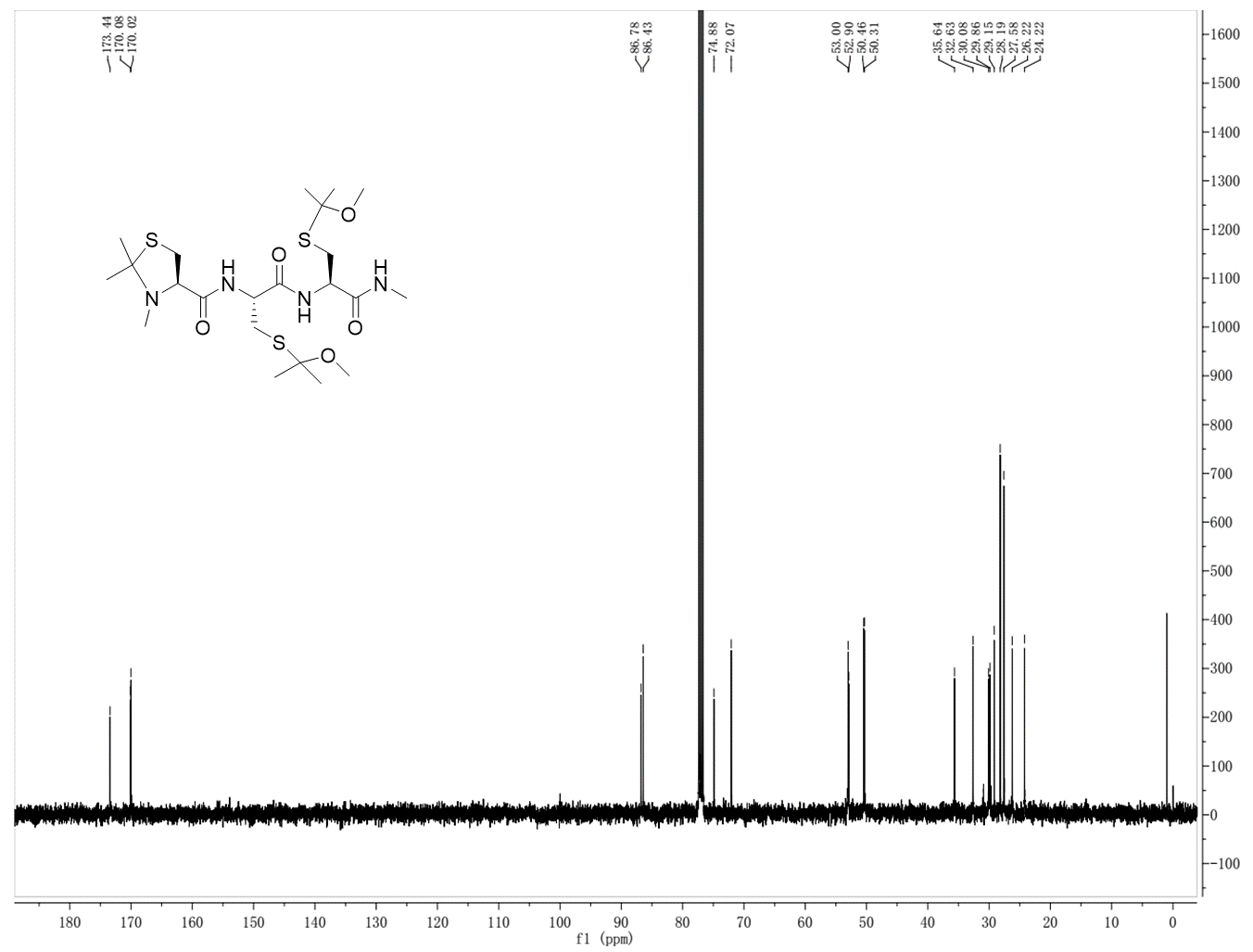


**Figure S14.** ^13^C NMR spectrum of compound **12**.


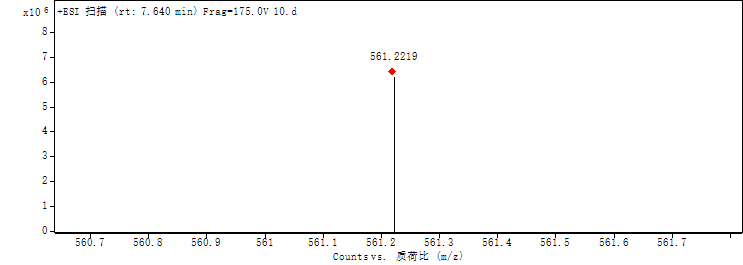


**Figure S15.** HRMS spectrum of compound **12**.

**
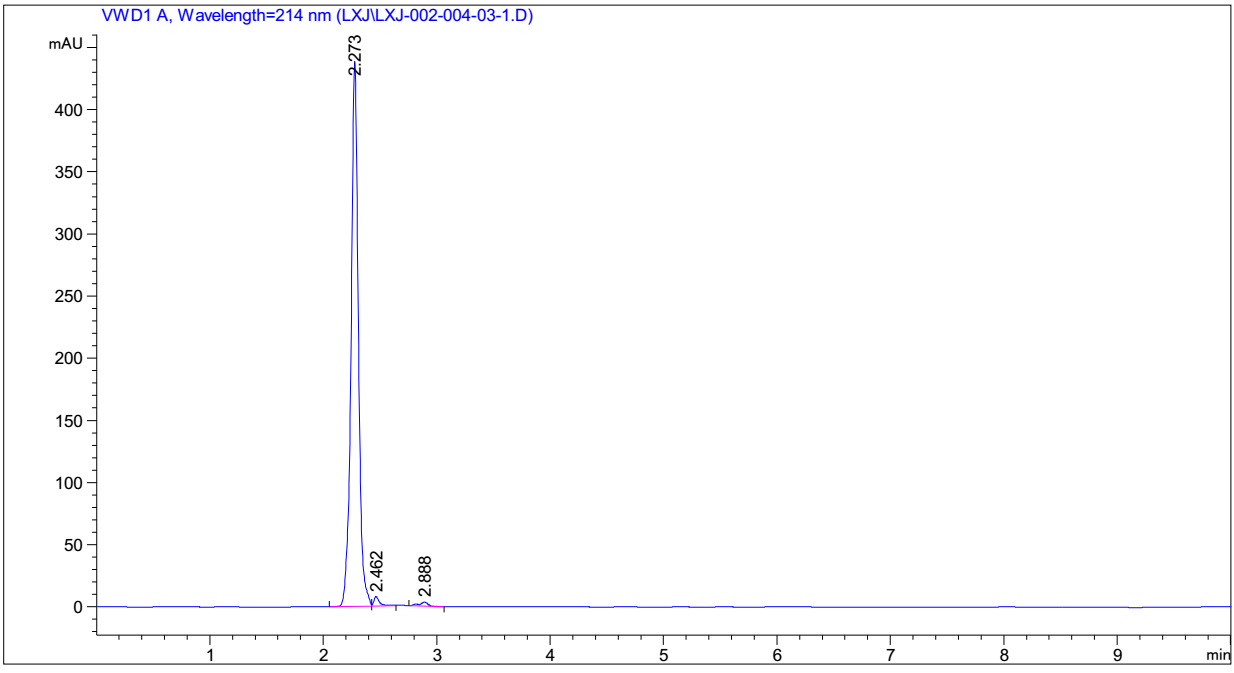
**

Wavelength = 214 nm

| Peak | RT (min) | Width  (min) | Area (mAU*s) | Height  (mAU) | Area (%) |
| --- | --- | --- | --- | --- | --- |
|  | 2.273 | 0.0687 | 1959.28760 | 434.12860 | 97.6684 |

**Figure S16.** HPLC spectrum of compound **12**.
